# Depression and anxiety among women with polycystic ovarian syndrome in low- and middle-income countries: a systematic review and meta-analysis

**DOI:** 10.3389/fgwh.2025.1688913

**Published:** 2025-11-25

**Authors:** Atimi Atinga, Hameed Akande Bashiru, Abiola Olajumoke Solomon, Oziegbe Oghide, Iyanu Adufe, Posi Emmanuel Aduroja, Adebukunola Olajumoke Afolabi, Ayobami Adebayo Bakare, Oluwaseyi Isaiah Olabisi, Philemon Paul Mshelia, Amaka Harry Ononuju, Amuchechukwu Veronica Nwafor, Ayokunmi Stephen Olusa, Oluchukwu Perpetual Okeke, Folahanmi Tomiwa Akinsolu, Olunike Rebecca Abodunrin, Olajide Odunayo Sobande

**Affiliations:** 1 Department of Public Health Technology, Taraba State College of Health Technology, Takum, Nigeria; 2 Department of Animal Sciences, Obafemi Awolowo University, Ile-Ife, Nigeria; 3 Department of Medical Biochemistry, Eko University of Medicine and Health Sciences, Lagos, Nigeria; 4 Accident & Emergency Department, Lagos University Teaching Hospital, Lagos, Nigeria; 5 Department of Public Health, Faculty of Basic Medical Sciences, Osun State University, Osun, Nigeria; 6 Department of Public Health, Faculty of Basic Medical and Health Sciences, Lead City University, Ibadan, Oyo, Nigeria; 7 Department of Nursing Science, Faculty of Basic Medical Sciences, College of Health Sciences, Obafemi Awolowo University, Ile-Ife, Nigeria; 8 Department of Community Medicine, University College Hospital, Ibadan, Oyo, Nigeria; 9 Department of Global Public Health, Karolinska Institutet, Stockholm, Sweden; 10 Department of Mental Health and Psychiatric Nursing, Faculty of Nursing Sciences, College of Health Science, Bowen University, Iwo, Nigeria; 11 Dept. of Physiology, Abubakar Tafawa Balewa University, Bauchi, Nigeria; 12 Department of Human Kinetics and Health Education, University of Nigeria Nsukka, Nsukka, Nigeria; 13 Department of Obstetrics and Gynaecology, Alex Ekwueme Federal University Ndufu-Alike, Abakaliki, Nigeria; 14 Department of Pharmacology, Faculty of Pharmacy, Obafemi Awolowo University, Ile-Ife, Nigeria; 15 Nigerian Institute of Medical Research Foundation, Yaba, Lagos, Nigeria; 16 Center for Reproduction and Population Studies, Clinical Sciences Department, Nigerian Institute of Medical Research, Yaba, Lagos, Nigeria; 17 Department of Research, Capacity Building and Implementation, Alabastron Initiative, Lagos, Nigeria; 18 Department of Epidemiology and Biostatics, Nanjing Medical University, Nanjing, China

**Keywords:** polycystic ovary syndrome, mental health disorder, psychological distress, meta-analysis, women health, endocrine disorder

## Abstract

**Background:**

Increasing evidence links Polycystic ovary syndrome (PCOS) with adverse mental health outcomes, particularly depression and anxiety. These challenges may be amplified in low- and middle-income countries (LMICs) due to limited awareness, restricted healthcare access, and sociocultural stigma.

**Objectives:**

To estimate the pooled prevalence of depression and anxiety among women of reproductive age with PCOS in LMICs and to examine clinical factors associated with these outcomes.

**Methods:**

Following PRISMA guidelines (PROSPERO CRD420251069068), we systematically searched PubMed, Scopus, Web of Science, and CINAHL for studies published between January 2005 and June 2025. Eligible studies included observational research reporting the prevalence of depression and/or anxiety in women aged 15–49 years with clinically diagnosed PCOS and assessed using validated tools. Data were pooled using a random-effects model. Subgroup and meta-regression analyses explored variations by study design, age, body mass index (BMI), country, and assessment tools. Heterogeneity was quantified with the I² statistic, and publication bias was assessed using funnel plots and Egger's test. Study quality was evaluated with the Joanna Briggs Institute checklist.

**Results:**

From 3,860 records, 40 studies met the inclusion criteria. All were rated low risk of bias (quality scores 75%–100%). The pooled prevalence of depression was 51% (95% CI: 43–59; I² = 97%), and anxiety was 45% (95% CI: 36–54; I² = 96%). The highest prevalence was observed among women aged 20–25 years (depression: 63%; anxiety: 56%) and in studies conducted in India (depression: 55%; anxiety: 51%). Clinical features such as infertility, hirsutism, and acne showed non-significant associations with depression or anxiety. No publication bias was detected.

**Conclusion:**

Depression and anxiety are highly prevalent among women with PCOS in LMICs, affecting nearly half of this population. These findings underscore the urgent need for integrating routine mental health screening and culturally tailored interventions into PCOS management in resource-limited settings.

**Systematic Review Registration:**

PROSPERO CRD420251069068.

## 1 Introduction

Polycystic Ovary Syndrome (PCOS) is the most common endocrine disorder in women of reproductive age, with a global prevalence estimated between 6% and 20% depending on diagnostic criteria used (1). It is characterized by a combination of androgen excess (e.g., hirsutism, acne), ovulatory dysfunction (e.g., irregular menses or anovulation), and polycystic ovarian morphology. The Rotterdam criteria remain the most widely accepted diagnostic standard, although definitions from the National Institute of Health (NIH) and Androgen Excess Society (AES) differ slightly in exclusions and thresholds (2, 3). The etiology of PCOS is multifactorial, involving both genetic predisposition and environmental influences such as sedentary lifestyles and poor dietary habits (4). While pharmacological, hormonal, and lifestyle interventions can manage symptoms, PCOS is incurable. However, it is associated with long-term complications, including type 2 diabetes, hypertension, infertility, and endometrial cancer, underscoring its significance as a global public health problem (5, 6).

PCOS is strongly associated with adverse mental health outcomes, particularly depression and anxiety (7). These associations are driven by the complex interaction of endocrine disruption, metabolic disturbances, chronic inflammation which may disrupt neurotransmitter function and mood regulation and psychosocial stressors (8, 9). Clinical features such as obesity, hirsutism, and infertility contribute to body image dissatisfaction and stigma, further exacerbating psychological distress (10). Several meta-analyses have quantified this burden globally. Cooney et al. (11) and Brutocao et al. (10) reported pooled prevalences of depressive and anxiety symptoms around 30%–40% in women with PCOS, while Dybciak et al. (12) found rates approaching 45% in mixed-income samples. However, most of these syntheses rely heavily on data from high-income settings, where diagnosis is earlier, awareness higher, and access to psychosocial care more robust. LMIC contexts differ substantially in ways that may amplify mental-health vulnerability among women with PCOS. Women in LMICs often face additional barriers, including delayed diagnosis, restricted access to mental health services, sociocultural expectations around fertility, and financial constraints, all of which may amplify psychological distress (13, 14). Stigma surrounding infertility and body hair, limited reproductive and psychiatric services, and delayed diagnosis due to weak health-system capacity may contribute to higher distress levels. Moreover, most studies in LMICs are facility-based, potentially underrepresenting women outside clinical care. These contextual differences suggest that the true prevalence of depression and anxiety in LMICs could exceed estimates from high-income countries, yet no prior meta-analysis has systematically quantified this. Emerging country-level data reinforce this concern. For instance, recent Indian studies have examined the psychosocial dimensions of PCOS: Kaur et al. (13, 15) identified menstrual irregularity, hirsutism, BMI, and age as significant predictors of poorer wellbeing. In addition, Jaswal et al. (16) found that only half of women in the Sub-Himalayan region demonstrated good knowledge of PCOS. Studies from Lebanon and Nigeria also highlight critical gaps in awareness and health-seeking behavior among young women with PCOS (14, 17). Therefore, this systematic review and meta-analysis aimed to determine the pooled prevalence of depression and anxiety among women of reproductive age with PCOS in LMICs. A secondary objective was to explore demographic, sociocultural, lifestyle, and clinical factors associated with these outcomes.

## 2 Methods

### 2.1 Study protocol registration and reporting

This systematic review and meta-analysis was conducted in accordance with the Preferred Reporting Items for Systematic Reviews and Meta-Analyses (PRISMA) 2020 guidelines (18). The study protocol was prospectively registered on 8th June, 2025 with the International Prospective Register of Systematic Reviews (PROSPERO) under the registration number CRD420251069068. The review focused specifically on studies conducted in LMICs, as defined by the World Bank per capita income classifications (19). This focus was chosen to address critical evidence gaps in resource-limited settings, where cultural stigma, limited awareness, and inadequate health infrastructure may amplify the mental health burden associated with PCOS. The PRISMA Checklist is presented in Supplementary File S1.

### 2.2 Review questions

The following review questions guided this systematic review and meta-analysis:

1. What is the prevalence of depression among women of reproductive age with PCOS in LMICs?
2. What is the prevalence of anxiety among women of reproductive age with PCOS in LMICs?
3. What are the clinical factors associated with depression and anxiety in this population?

### 2.3 Review framework (PECO)

Table 1 outlines the PECO framework (Population, Exposure, Comparator/Context, Outcome) guiding the review. It specifies that the study population is women of reproductive age (15–49 years) with clinically diagnosed PCOS in LMICs. The exposure is PCOS, defined by its clinical features infertility, obesity, and hirsutism which were assessed using standardized diagnostic and anthropometric criteria reported in the included studies. The outcomes of interest are primarily the prevalence of depression and anxiety measured with validated tools, with secondary outcomes focusing on clinical factors linked to these mental health conditions.

**Table 1:** PECO framework for the review on depression and anxiety in women with PCOS in LMICs.

Component	Description
Population (P)	Women of reproductive age (15–49 years) clinically diagnosed with PCOS using standardized criteria (Rotterdam, NIH, AES) in LMICs.
Exposure (E)	Diagnosis of PCOS and associated clinical features (e.g., infertility, hirsutism, acne, menstrual irregularities, obesity).
Comparator/Context (C)	Women without PCOS (for case-control studies) and studies conducted in low- and middle-income countries (LMICs) across clinical, community, or population-based settings.
Outcome (O)	Primary outcomes: Prevalence of depression and anxiety measured with validated assessment tools (e.g., PHQ-9, BDI, GAD-7, HADS, STAI). Secondary outcomes: Demographic, sociocultural, lifestyle, and clinical factors associated with depression and anxiety.

### 2.4 Eligibility criteria

Studies were considered eligible if the following conditions were met:The study population comprised women of reproductive age (15–49 years) who were clinically diagnosed with PCOS based on established diagnostic criteria such as the Rotterdam (20), National Institutes of Health (21), or Androgen Excess Society (22) definitions. Eligible studies were also required to assess depression and/or anxiety using standardized tools such as the Patient Health Questionnaire-9 (PHQ-9), Beck Depression Inventory-II (BDI-II), Hospital Anxiety and Depression Scale (HADS), or Generalized Anxiety Disorder-7 (GAD-7). Only studies conducted in LMICs, as defined by the World Bank 2024 classification, were included (19). Studies had to report the prevalence of depression and/or anxiety among women with PCOS or provide sufficient data to allow calculation of prevalence. Observational designs, including cross-sectional, prospective or retrospective cohort, and case–control studies, were eligible. However, for eligible case-control studies, only data from their baseline or PCOS sample arms were extracted for prevalence estimation.

Studies were excluded if PCOS was self-reported without clinical confirmation. Studies were also excluded if participants were primarily diagnosed with depression or anxiety and PCOS was considered only as a comorbidity. Similarly, studies that assessed depressive symptoms solely in relation to individual PCOS manifestations such as obesity, infertility, or hirsutism were not eligible. In terms of population, studies involving pregnant or postmenopausal women with PCOS, as well as those conducted outside LMICs or published prior to 2005, were excluded. With respect to study design, randomized controlled trials (RCTs), quasi-RCTs, crossover trials, controlled before–and–after studies, systematic reviews, meta-analyses, case series, reviews, commentaries, expert opinions, editorials, conference proceedings, letters, and study protocols were all excluded.

### 2.5 Search strategy

A comprehensive search of four international databases, PubMed, Scopus, CINAHL, and Web of Science, was performed to identify eligible studies published between January 1, 2005, and June 16, 2025. The lower year limit was chosen to capture studies conducted after the widespread adoption of the Rotterdam criteria for PCOS diagnosis in 2005. The search focused on depression and anxiety among women of reproductive age with PCOS in LMICs. The strategy combined controlled vocabulary (e.g., MeSH terms) and free-text keywords related to polycystic ovary syndrome and mental health outcomes. Search terms for PCOS included “*Polycystic ovarian syndrome,” “Polycystic ovary syndrome,” “PCOS,” “Stein-Leventhal syndrome,” “Sclerocystic ovary syndrome,” “Polycystic ovarian disease,”* and related variants. Mental health terms included “*depression,” “anxiety,” “mood disorders,” “psychological distress,”* and “*mental health.”* Boolean operators “AND” and “OR” were applied to combine terms appropriately.

No restrictions were placed on language or publication status. The full search strategies including the date of last search for each database are provided in Supplementary File S2.

### 2.6 Study selection

All retrieved records were imported directly into Rayyan (23), which was used for the entire screening process, including automatic de-duplication and blinded screening. Two reviewers (HAB and OO) independently screened titles and abstracts for relevance based on the eligibility criteria. Full-text articles of potentially eligible studies were then screened independently by two additional reviewers (AOS and AA). Any conflicts were resolved through discussion, with arbitration by a third reviewer (IA) when necessary. (See Supplementary File S3).

### 2.7 Data extraction

Data were extracted using a piloted extraction form to ensure consistency and replicability across studies. For each eligible study, we recorded the author's name, year of publication, study location, and survey period. Key study characteristics such as design, sample size, and participant demographics, including age distribution and mean BMI, were documented. Information on the diagnostic criteria used for PCOS (e.g., Rotterdam, NIH, AES) and the specific instruments employed to assess depression and anxiety (e.g., PHQ-9, BDI-II, HADS, GAD-7) was also collected.

The primary outcomes extracted were the prevalence of depression and anxiety among women with PCOS, along with the corresponding number of cases and total sample sizes. Where available, we also extracted odds ratios (ORs) with 95% confidence intervals (CIs) for associated risk factors such as menstrual irregularity, infertility, and hirsutism. When multiple estimates were reported, preference was given to the most fully adjusted models. Data extraction was performed independently by two reviewers, and all discrepancies were resolved through discussion and consensus. The final dataset was used to conduct subgroup analyses (See Supplementary File S4).

### 2.8 Quality appraisal

The methodological quality of all included studies was assessed using the Joanna Briggs Institute (JBI) Critical Appraisal Checklist appropriate to each study design (24). Each study was scored against the checklist, and overall quality was categorized as low risk of bias (score ≥6), moderate risk of bias (score 4–5), or high risk of bias (score ≤4). Two reviewers (OO and IA) conducted the assessment independently, and any disagreements were resolved through a third reviewer (HAB). Inter-rater reliability for overall quality ratings was assessed using Cohen's kappa (*κ* = 0.82, 95% CI 0.76–0.88), indicating substantial agreement. Of the 40 studies included in this review, all met the quality criteria. For comparability, we converted raw scores to percentage scores and categorized overall risk of bias as low (≥75%), moderate (50%–74%), or high (<50%) (See Supplementary File S5).

### 2.9 Data analysis

All statistical analyses were performed using R Studio version 4.4.1 with the *meta*, *metafor*, and *loo* packages. Prevalence estimates for depression and anxiety among women with PCOS were pooled using a random-effects model (REM). This model was selected because it accounts for both within-study and between-study variability, thereby providing more conservative and generalizable estimates under the assumption that true effect sizes may vary across studies. Odds ratios (ORs) with 95% confidence intervals (CIs) were also pooled to evaluate associations between clinical risk factors (e.g., infertility, hirsutism) and mental health outcomes. To explore sources of variability, subgroup analyses were conducted according to study location, study design, participant age group, sample size, BMI category, and type of depression or anxiety assessment tool used. Heterogeneity was assessed using Cochran's *Q* test and quantified with the I² statistic, which expresses the percentage of variability due to true heterogeneity rather than chance. Consistent with established thresholds, I² values of approximately 25%, 50%, and 75% were interpreted as low, moderate, and high heterogeneity, respectively. The robustness of findings was further assessed through sensitivity analyses, including a leave-one-out approach, which sequentially removes individual studies to evaluate their influence on the pooled estimates. To evaluate potential publication bias, funnel plots were visually inspected for asymmetry while Egger's and Begg's statistical tests were applied to formally test for bias. In addition, the trim-and-fill method was used to estimate the potential impact of unpublished or missing studies and provide corrected pooled estimates. This approach enhances the reliability of the findings by accounting for small-study effects and selective reporting. Statistical significance was defined at *p* < 0.05 for all analyses.

### 2.10 GRADE assessment

The certainty of evidence for depression and anxiety outcomes, as well as for subgroup determinants and associated clinical features, was evaluated using the Grading of Recommendations Assessment, Development, and Evaluation (GRADE) approach (25, 26). Under the GRADE framework, the body of evidence derived from observational studies was initially rated as low certainty, but the overall rating could be downgraded or upgraded depending on specific methodological considerations.

Evidence was downgraded in situations where serious concerns were identified. This included the risk of bias due to limitations in study design or reporting; inconsistency, reflected by substantial heterogeneity across studies (I² > 50%); and indirectness were outcomes were measured using surrogate tools or limited to specific populations. Imprecision was also noted, arising from small sample sizes or wide confidence intervals. Finally, potential publication bias was assessed using funnel plot asymmetry and Egger's regression test.

Following this structured process, each outcome was assigned a final rating of high, moderate, low, or very low certainty. To enhance transparency, a Summary of Findings (SoF) table was prepared following Cochrane guidance and RevMan conventions (27). This table presents the pooled effect estimates, number of participants, degree of heterogeneity, and corresponding certainty ratings for each outcome, allowing readers to critically appraise the strength of evidence generated by this review. (See Supplementary File S6).

## 3 Results

### 3.1 Study selection

The systematic search identified a total of 3,858 records across the four databases (PubMed = 861, Scopus = 1,455, Web of Science = 1,268, CINAHL = 272), along with two (2) additional records identified through supplementary sources. Following the removal of 1,955 duplicates, 1,903 unique records were screened by title and abstract. Of these, 1,853 were excluded for not meeting eligibility criteria.

The remaining 50 full-text articles were retrieved and assessed in detail. Ten (10) were subsequently excluded; seven (7) because they did not report prevalence estimates for depression or anxiety (28–34), and three (3) because they relied on non-validated assessment tools (35–37). Ultimately, 40 studies fulfilled the inclusion criteria and were incorporated into both the systematic review and the meta-analysis (13, 38–76).

**Figure 1:**
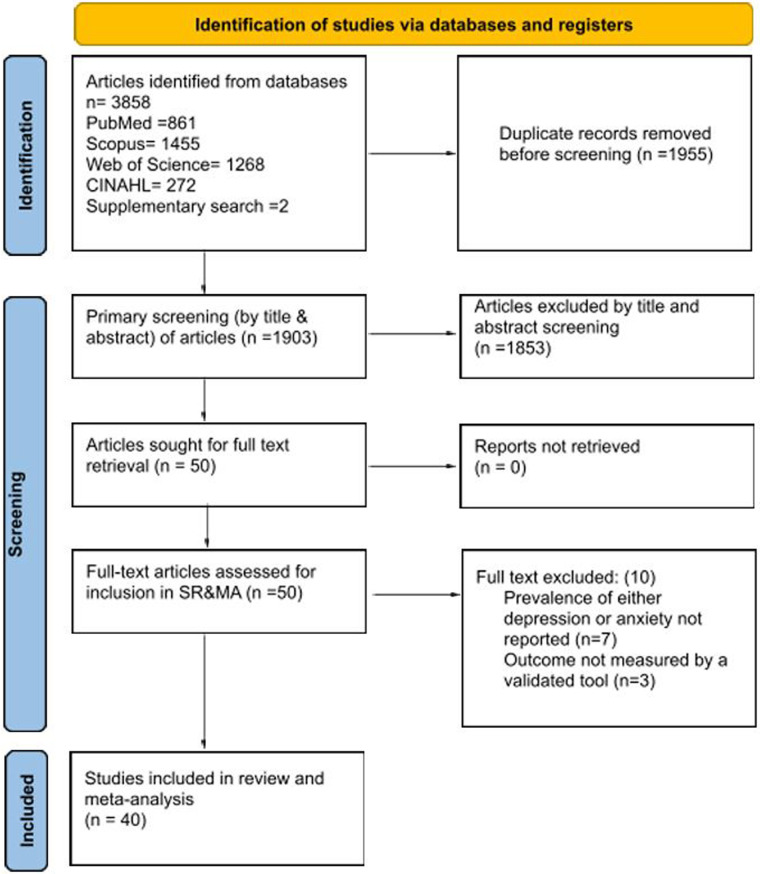
PRISMA flow diagram.

The full process of study identification, screening, eligibility assessment, and inclusion is illustrated in the PRISMA 2020 flow diagram (Figure 1).

### 3.2 Study characteristics

The systematic review included 40 studies published between 2009 and 2025, representing a total of 6,411 women of reproductive age with PCOS across LMICs in South Asia (*n* = 22) (13, 39, 41, 44, 46, 47, 51–53, 55, 56, 59, 63–69, 71, 73, 74), South East Asia (*n* = 1) (48), the Middle East (*n* = 13) (38, 40, 42, 43, 45, 49, 54, 57, 61, 70, 72, 75, 76), South America (*n* = 3) (58, 60, 62) and Africa (*n* = 1) (50). Most of the studies were conducted in Iran (*n* = 13) (38, 40, 42, 43, 45, 49, 54, 57, 61, 70, 72, 75, 76), Pakistan (*n* = 9) (39, 44, 51, 56, 63, 68, 69, 73, 74), India (*n* = 11) (13, 41, 46, 47, 52, 55, 59, 64–67), Bangladesh (*n* = 2) (53, 71), Brazil (*n* = 3) (58, 60, 62), Egypt (*n* = 1) (50), and the Philippines (*n* = 1) (48).

Sample sizes varied considerably, ranging from 27 participants (73) to 742 participants (72). Participant ages were generally within the reproductive years (15–49 years), with mean ages reported between 21.4 and 32.1 years. Where available, mean BMI values ranged from 21.8 kg/m² to 33.6 kg/m², spanning normal weight to obese categories.

Most studies assessed depression and anxiety(*n* = 28) (13, 40, 41, 43–45, 47, 48, 51–55, 57–60, 62, 63, 65, 66, 69–73, 75, 76), while a smaller number focused on a single outcome, depression(*n* = 9) (38, 42, 46, 49, 50, 56, 61, 64, 74) and anxiety (*n* = 3) (39, 67, 68). A wide range of validated screening tools were employed.

For depression, the most used instruments included the BDI (*n* = 8) (38, 40, 42, 49, 50, 55, 61, 75), HADS-D (*n* = 12) (43–45, 48, 51, 57, 58, 60, 62, 65, 69, 76), and the PHQ-9 (*n* = 4) (46, 50, 64, 72), alongside others such as HDRS (*n* = 4) (13, 47, 66, 70), DASS-21 (*n* = 4) (41, 53, 63, 73), and QIDS-SR (*n* = 1) (56). For anxiety, frequently applied measures included the HADS-A (*n* = 9) (43–45, 48, 51, 57, 60, 69, 76), HAM-A (*n* = 5) (47, 55, 65, 66, 70), DASS-21 (*n* = 6) (41, 53, 63, 67, 71, 73), BAI (*n* = 3) (40, 58, 75), and GAD-7 (*n* = 1) (39).

A detailed summary of study characteristics, including country, sample size, participant demographics, diagnostic criteria, screening tools, and prevalence estimates, is presented in Table 2.

**Table 2:** Characteristics of the studies included in the systematic review and meta-analysis of depression and anxiety in women of reproductive age living with PCOS in LMICs.

Study	Country	Sample Size	Age Range (years)	Mean Age	BMI (Mean)	Mental Health Outcomes Assessed	Assessment Tool Used		Prevalence (%)	
							Depression	Anxiety	Depression	Anxiety
Aliasghari et al. (38)	Iran	174	15–49	32.0	26.5	Depression	BDI	–	91.2	–
Asghar et al. (39)	Pakistan	105	15–40	28.1	21.8	Anxiety	–	GAD-7	–	25
Bahadori et al. (40)	Iran	239	20–43	30.42	26.46	Both	BDI	BAI	74	30.4
Bansal et al. (41)	India	104	18–45	31.5	26.06	Both	DASS-21	DASS-21	46.16	64.42
Basirat et al. (43)	Iran	135	18–45	30.8		Both	HADS	HADS-A	33.9	33.1
Basirat et al. (42)	Iran	120	15–45	29.55	27.26	Depression	BDI	–	73.9	–
Batool et al., (44)	Pakistan	137	18–45	25	33.6	Both	HADS	HADS	30	15
Bazarganipour et al. (45)	Iran	300	15–40	26.56	25.87	Both	HADS	HADS	5	32
Bhattacharya and Jha, (46)	India	117	16–35	21.9	26.3	Depression	PHQ	–	64.1	–
Chaudhari et al. (47)	India	70	18–45	27.65		Both	HDRS	HAM-A	25.7	38.6
Cupino-Arcinue et al. (48)	Phillipines	253	18–40	28	25.02	Both	HADS	HADS	9.09	46.25
Enjezab et al. (49)	Iran	62	20–40	29.97	–	Depression	BDI	–	64.5	–
Gomaa et al. (50)	Egypt	30	18–45	27.2	32.24	Depression	BDI	–	53.33	–
Habib et al. (51)	Pakistan	140	20–40	30		Both	HADS	HADS	70.7	72.9
Halder et al. (52)	India	189	15–49	32.09	24.3	Both	PHQ	PHQ	52.4	45
Haseen et al. (53)	Bangdalesh	266	18–40	24.5	–	Both	DASS-21	DASS-21	85.3	96.9
Hemmatadadi et al. (54)	Iran	69	20–45	30.79	26.98	Both	SCL-90	SCL-90	33.3	15.9
Hosseini et al. (76)	Iran	470	18–45	28.72	23.72	both	HADS	HADS-D	52.8	59.8
Joshi et al., (55)	India	105	18–37	25.1		Both	BDI	HAM-A	51.4	47.6
Kanwal et al. (56)	Pakistan	60	18–45	27.86	28.56	Depression	QIDS-SR	–	55	–
Kaur et al. (13)	India	60	18–40	29		Both	HDRS	HDRS	61	57
Kazemi et al. (57)	IRAN	150	18–30	22.78	22.36	Both	HADS	HADS	54.7	53.3
Kogure et al. (58)	Brazil	96	18–39	28.8	29.1	both	HADS	BAI	32.1	57.1
Korampatta et al. (59)	India	50	18–40	26.68	–	Both	SCL-90-R	SCL-90-R	60.6	34
Lara et al. (60)	Brazil	43	18–37	27.8	27.91	Both	HADS	HADS	34.8	44.1
Mirghafourvand et al. (61)	Iran	174	15–49	28.9	26.73	Depression	BDI	–	98.2	–
Moreira et al. (62)	Brazil	109	14 −40	26.8		Both	HADS	Mental Health subscale	80	–
Mughal et al. (63)	Pakistan	100	20–40	30		Both	DASS-21	DASS-21	75	91
Mukundan & Jayakumari, (64)	India	186	15–35	23.38		Depression	PHQ	–	76.9	–
Nayar et al. (65)	India	120	15–49	32	–	Both	HADS	HAM-A	37.5	41.67
Nidhi et al. (67)	India	150	15–49	28	28.7	Anxiety	–	DASS-21	–	66.7
Prathap et al. (66)	India	64	15–35	25		Both	HDRS	HAM-A	93	100
Rafique & Ilyas, (68)	Pakistan	180	19–24	21.4		Anxiety	–	SIAS		25
Rizwan Khan et al. (69)	Pakistan	74	18–40	26.8	28.7	Both	HADS	HADS	17.6	20.3
Salehifar et al. (70)	India	50	18–35	23.48		Both	HDRS	HAM-A	54	64
Salma et al. (71)	Bangdalesh	240	19–40	25.2	27.9	Both	DASS-21	DASS-21	47.5	44.2
Sayyah-Melli et al. (72)	Iran	742	18–35	23.5	27.4	Both	PHQ	PHQ	18.9	7.7
Siddique et al. (73)	Pakistan	27	18–25	21.4	–	Both	DASS-21	DASS-21	59.2	22.4
Sidra et al. (74)	Pakistan	440	15–44	29.5		Depression	SF-12	–	61.8	–
Zangeneh et al. (75)	Iran	201	18–45	30		Both	BDI	BAI	32.3	45.8

### 3.3 Risk of bias

The methodological quality of the included studies was assessed using the JBI critical appraisal checklist. Overall, the quality of evidence was strong: all 40 studies were rated as low risk of bias, with scores ranging from 75% to 100%. More than half of the studies (*n* = 18; 45%) scored 75% (38–40, 42, 43, 49, 51, 55, 59, 66, 68–70, 73–76), while 11 studies (27.5%) achieved 87.5% (47, 50, 53, 54, 56, 58, 60, 63, 64, 71, 72). In addition, 11 studies (17.5%) also obtained a perfect score of 100% (13, 41, 44, 46, 48, 52, 57, 61, 62, 65, 67). Importantly, no study was classified as high risk of bias (See Supplementary File S5).

### 3.4 Meta-analysis for pooled prevalence of depression and anxiety

**Figure 2:**
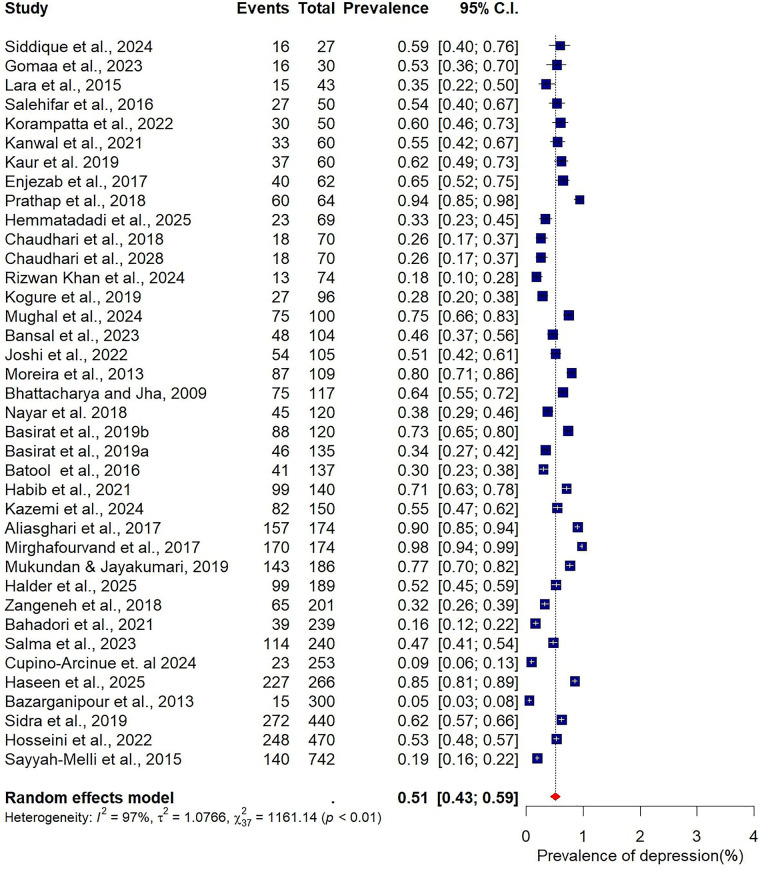
Forest plot of pooled prevalence of depression in women of reproductive Age with PCOS in Low- and middle-income countries.

**Figure 3:**
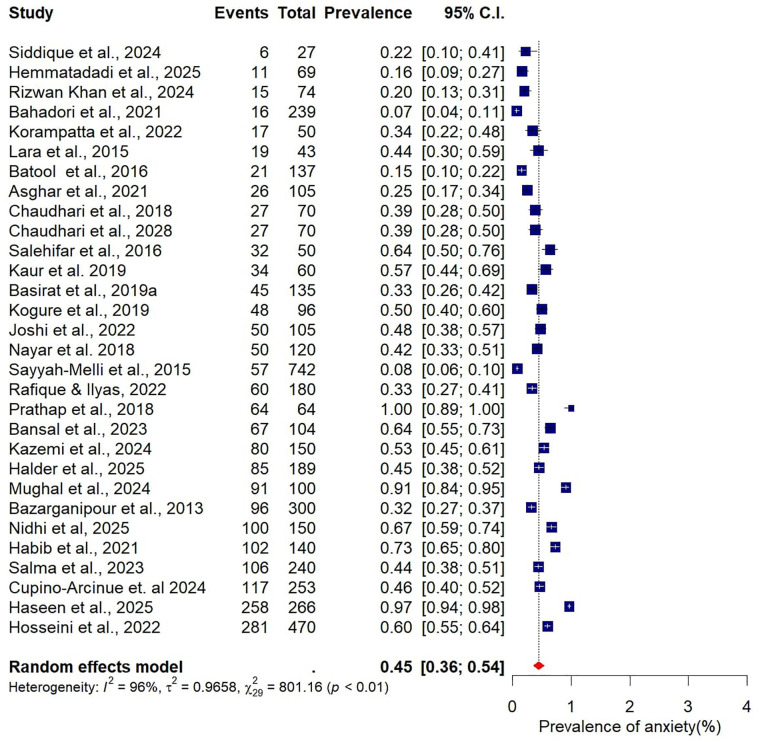
Forest plot of pooled prevalence of anxiety in women of reproductive Age with PCOS in Low- and middle-income countries.

The pooled prevalence estimates for depression and anxiety among women of reproductive age with PCOS in LMICs are presented in Figures 2,3. A total of 38 studies contributed data on depression, while 30 studies reported on anxiety. The meta-analysis revealed that the prevalence of depression was 51% (95% CI: 43%–59%), indicating that approximately one in two women with PCOS in LMICs experience clinically significant depressive symptoms. The pooled prevalence of anxiety was 45% (95% CI: 36%–54%), suggesting that nearly half of this population also report anxiety symptoms. For both conditions, substantial heterogeneity was observed (I² = 97% for depression; I² = 96% for anxiety; *p* < 0.01).

### 3.5 Subgroup analysis

Given the high heterogeneity observed in the overall pooled estimates of depression and anxiety (I² > 95%), these analyses stratified studies by participant characteristics (age, BMI), methodological features (study design, sample size, assessment tool), and geographic setting (country).

#### 3.5.1 Depression

**Figure 4:**
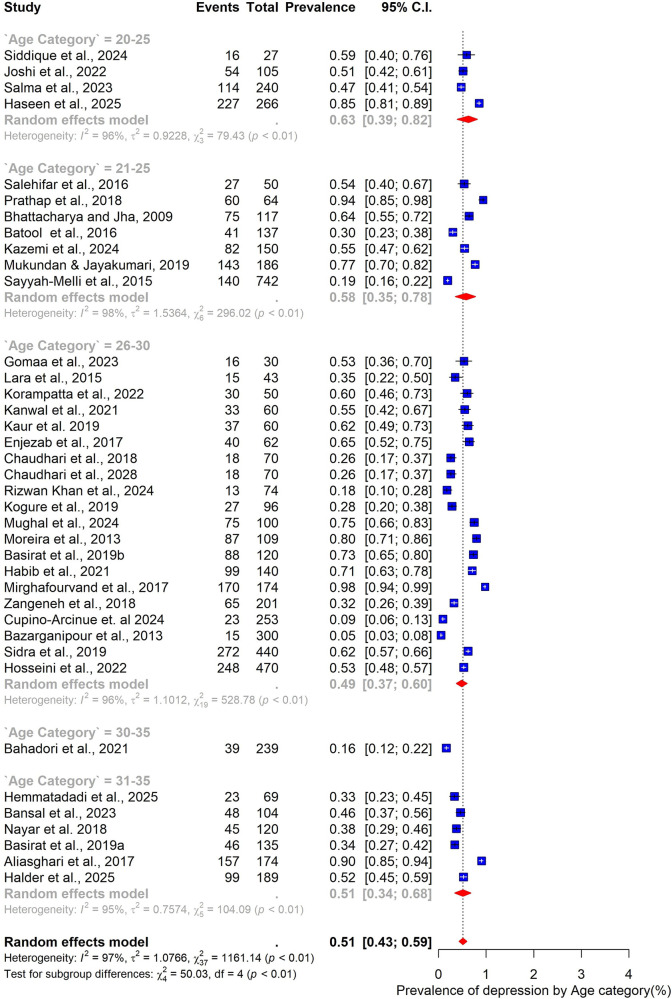
Subgroup analysis of depression prevalence in women with PCOS by Age category.

**Figure 5:**
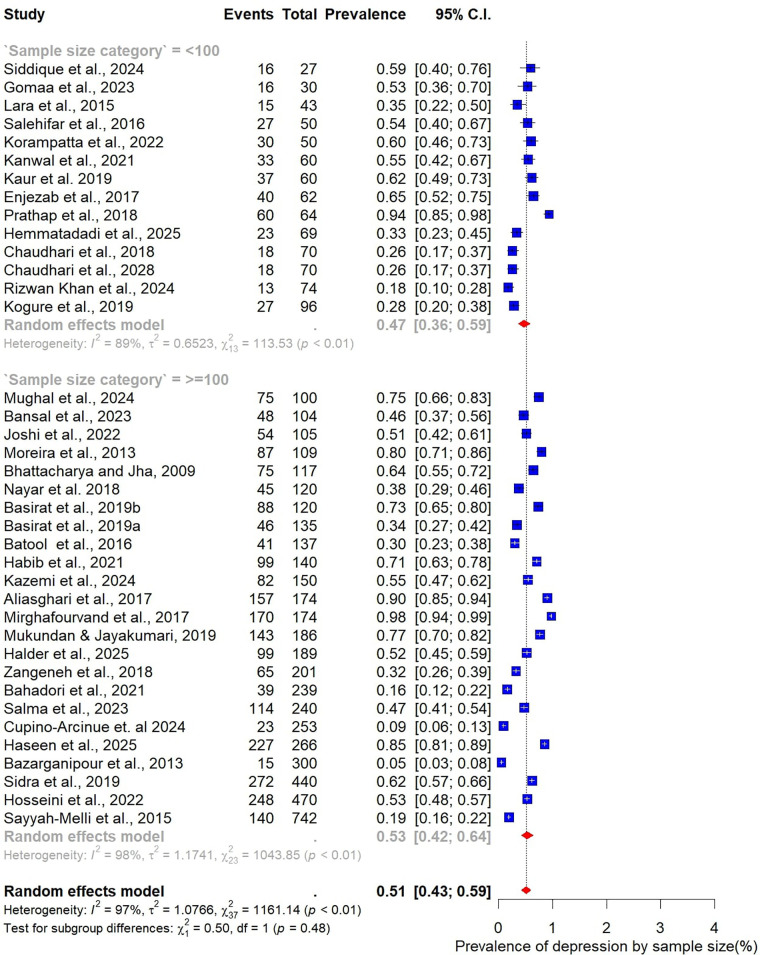
Subgroup analysis of depression prevalence in women with PCOS by sample size category.

**Figure 6:**
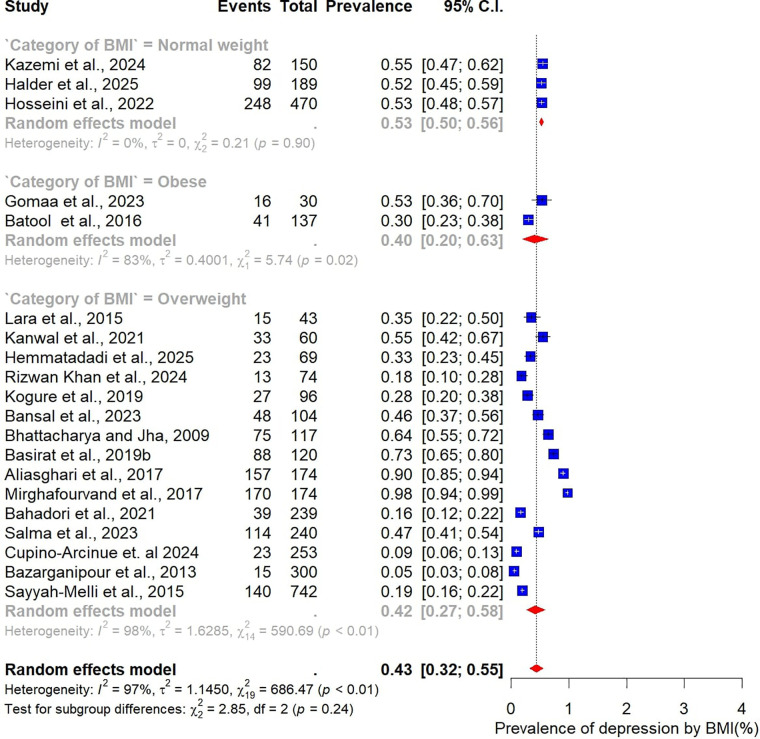
Subgroup analysis of depression prevalence in women with PCOS by BMI category.

**Figure 7:**
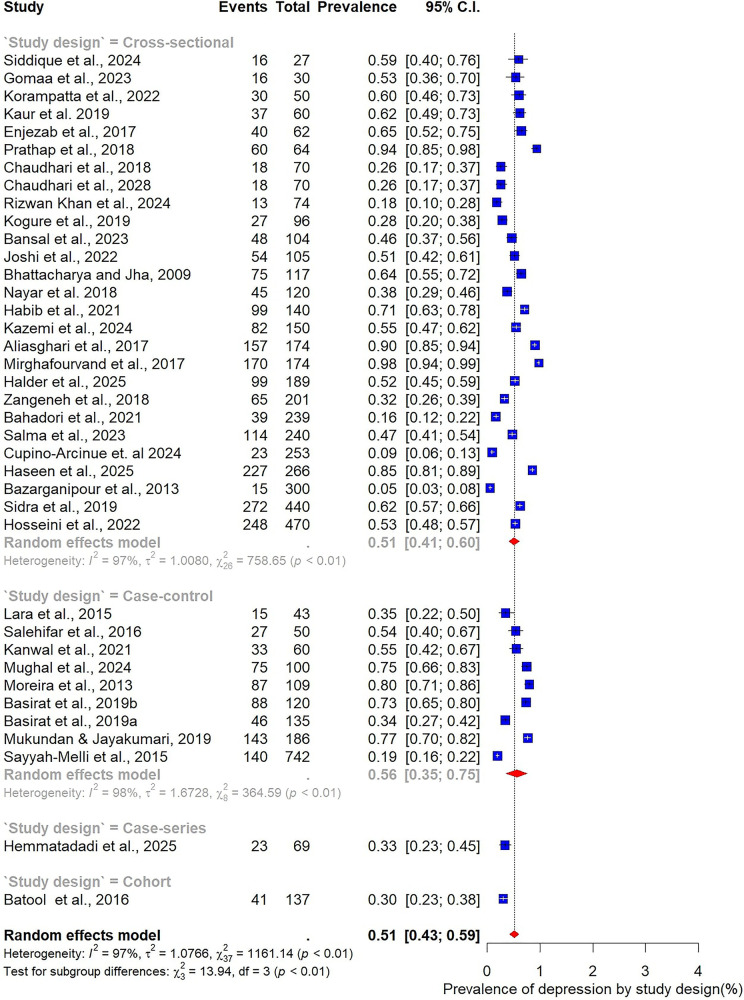
Subgroup analysis of depression prevalence in women with PCOS by study design.

**Figure 8:**
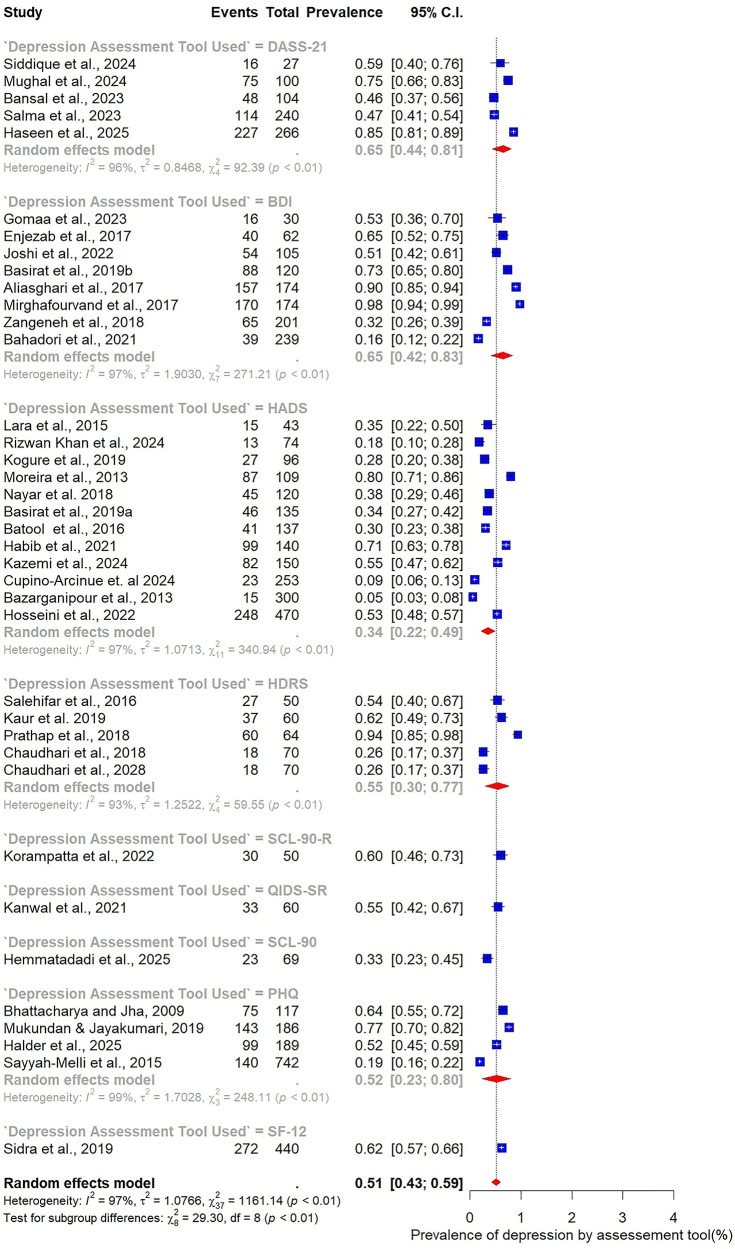
Subgroup analysis of depression prevalence in women with PCOS by assessment tool.

**Figure 9:**
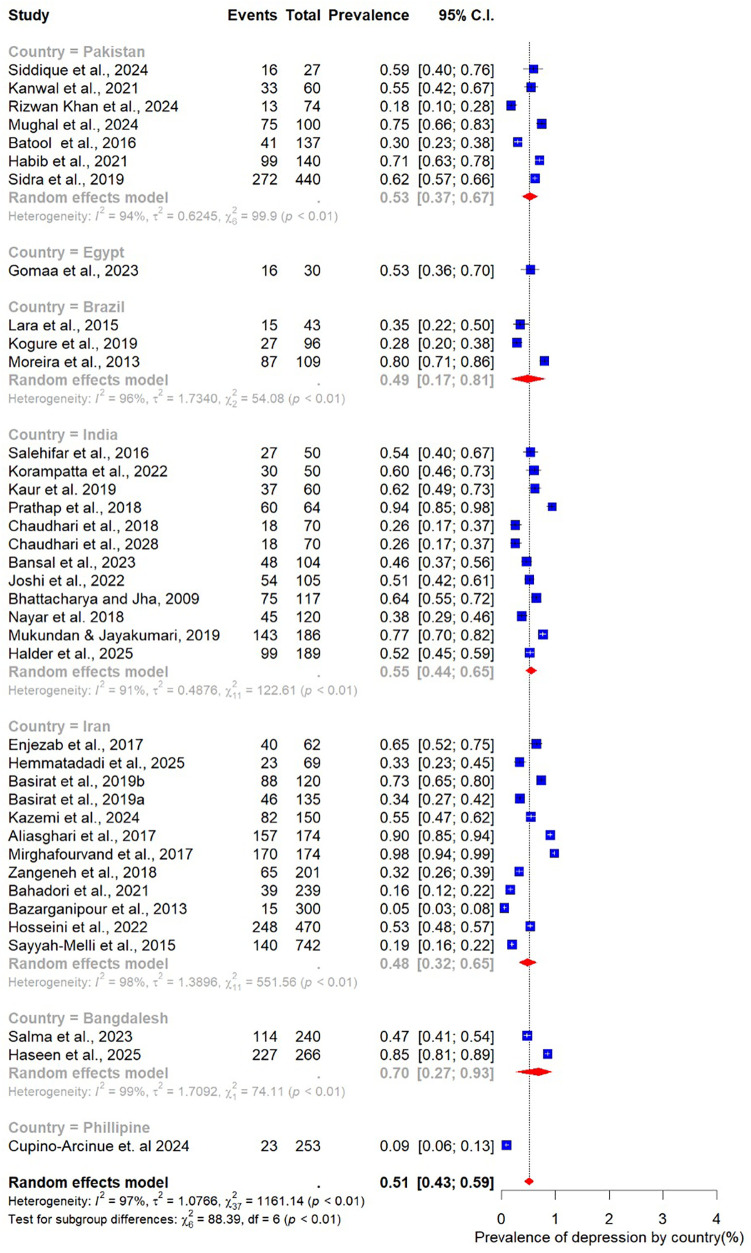
Subgroup analysis of depression prevalence in women with PCOS by country.

Subgroup analyses were conducted to explore how the prevalence of depression among women with PCOS varied across participant characteristics, study design, and methodological factors (Figures 4–9).

When stratified by age group, the highest prevalence was observed among younger women. Those aged 20–25 years had a pooled prevalence of 63% (95% CI: 39–82; *n* = 4), while those in the 21–25-year group also showed a high prevalence of 58% (95% CI: 35–78; *n* = 7). The 26–30-year age group, which accounted for most included studies (*n* = 20), demonstrated a somewhat lower prevalence of 49% (95% CI: 37–60). Women aged 31–35 years had a prevalence of 51% (95% CI: 34–68; *n* = 6) (Figure 4).

Analysis by sample size revealed slightly higher prevalence estimates in larger studies. Studies with fewer than 100 participants (*n* = 14) reported a pooled prevalence of 47% (95% CI: 36–59), while those with 100 or more participants (*n* = 24) yielded a higher prevalence of 53% (95% CI: 42–64) (Figure 5). When stratified by BMI, differences were also evident. In the three studies restricted to normal-weight women, the pooled prevalence of depression was 53% (95% CI: 50–56), with no observed heterogeneity (I² = 0%). In contrast, 15 studies focusing on overweight women reported a lower pooled prevalence of 42% (95% CI: 32–55), accompanied by substantial heterogeneity (Figure 6).

Subgroup analysis by study design showed broadly similar results across designs. Among the 27 cross-sectional studies, the pooled prevalence of depression was 51% (95% CI: 41–60; I² = 97%). In comparison, the nine case–control studies reported a slightly higher prevalence of 56% (95% CI: 35–75; I² = 98%), indicating that methodological differences may contribute only modestly to variability (Figure 7).

Marked variability was observed in relation to the assessment tool used. The HADS, employed in 12 studies, yielded the lowest prevalence estimate at 34% (95% CI: 22–49). In contrast, studies using the BDI (*n* = 8) reported substantially higher prevalence at 65% (95% CI: 42–83). Other commonly applied tools also demonstrated elevated estimates, including the DASS-21 (65%, 95% CI: 44–81), the HDRS (55%, 95% CI: 30–77), and the Patient Health Questionnaire (PHQ) (52%, 95% CI: 23–80) (Figure 8).

Finally, geographic variation was evident. Studies conducted in India (*n* = 12) reported the highest pooled prevalence at 55% (95% CI: 44–55), closely followed by Pakistan at 53% (95% CI: 37–67; *n* = 7). In Iran, the prevalence was slightly lower at 48% (95% CI: 32–65; *n* = 6) (Figure 9).

#### 3.5.2 Anxiety

**Figure 10:**
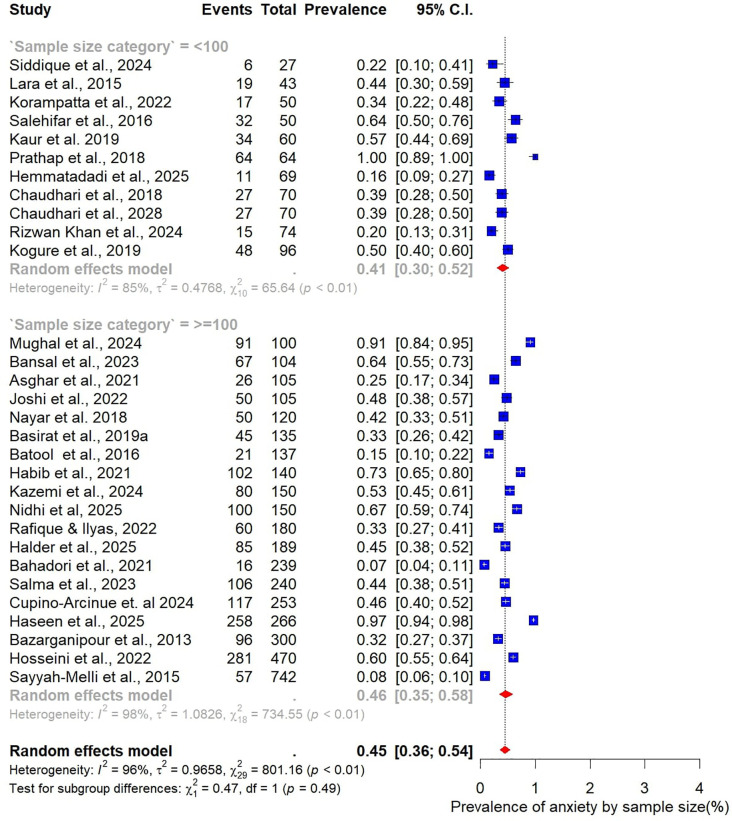
Subgroup analysis of anxiety prevalence in women with PCOS by study sample size.

**Figure 11:**
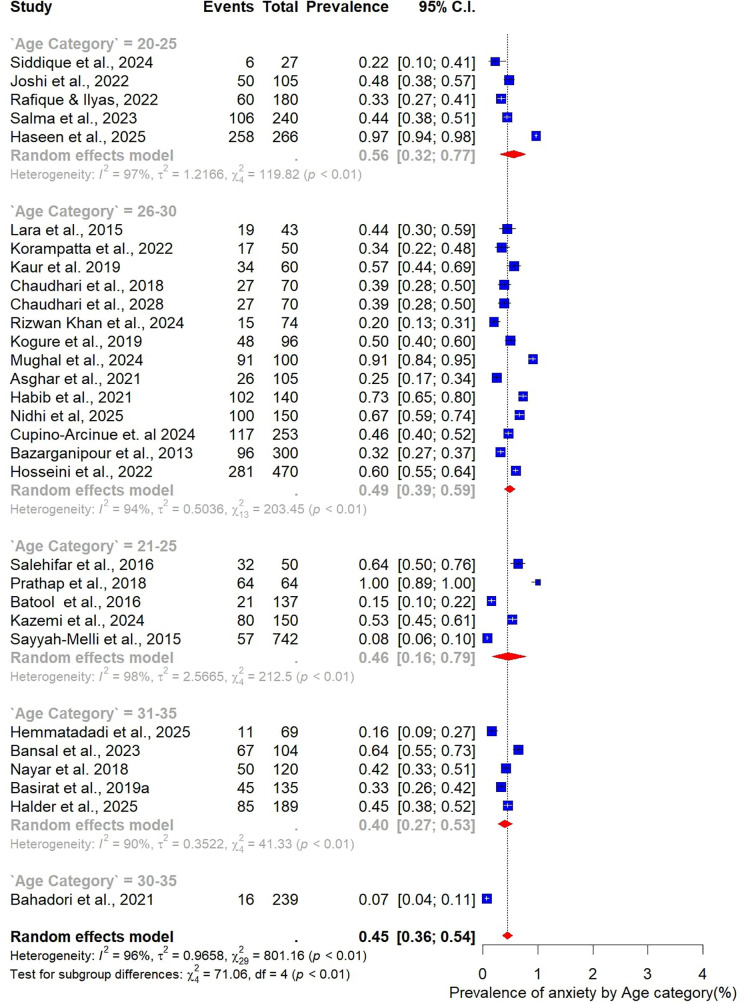
Subgroup analysis of anxiety prevalence in women with PCOS by Age category.

**Figure 12:**
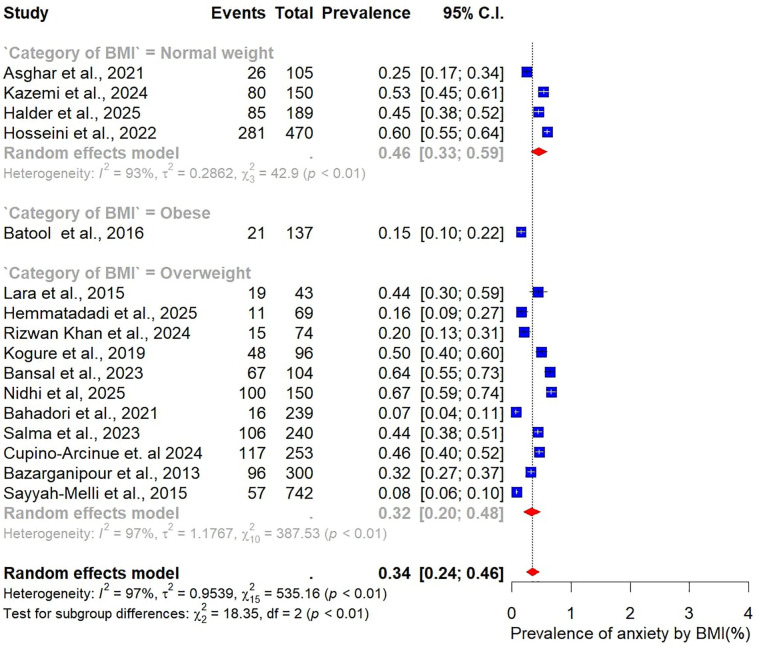
Subgroup analysis of anxiety prevalence in women with PCOS by BMI category.

**Figure 13:**
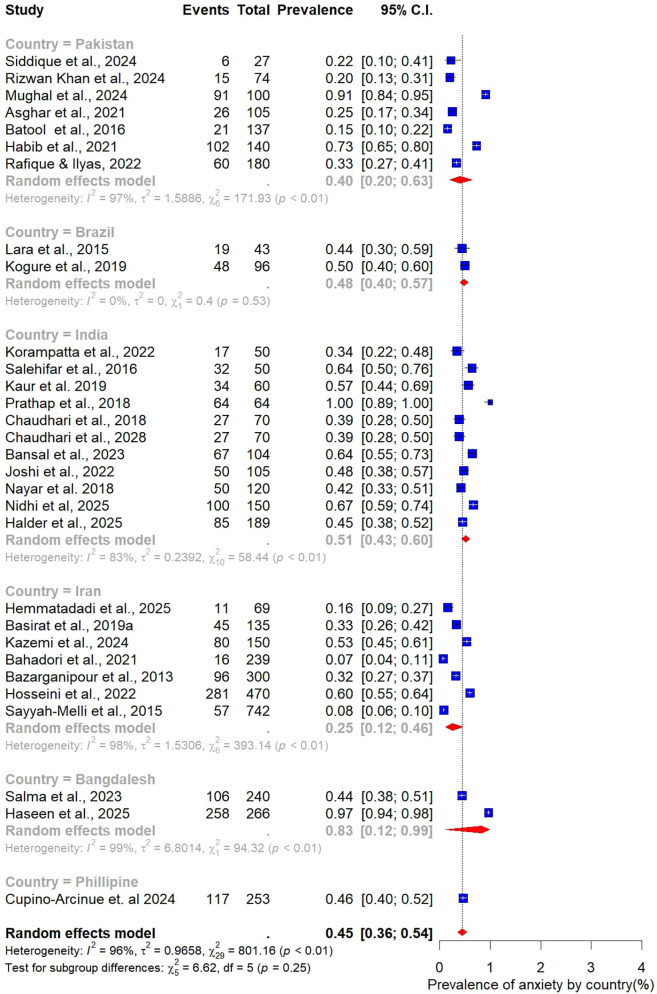
Subgroup analysis of anxiety prevalence in women with PCOS by country.

**Figure 14:**
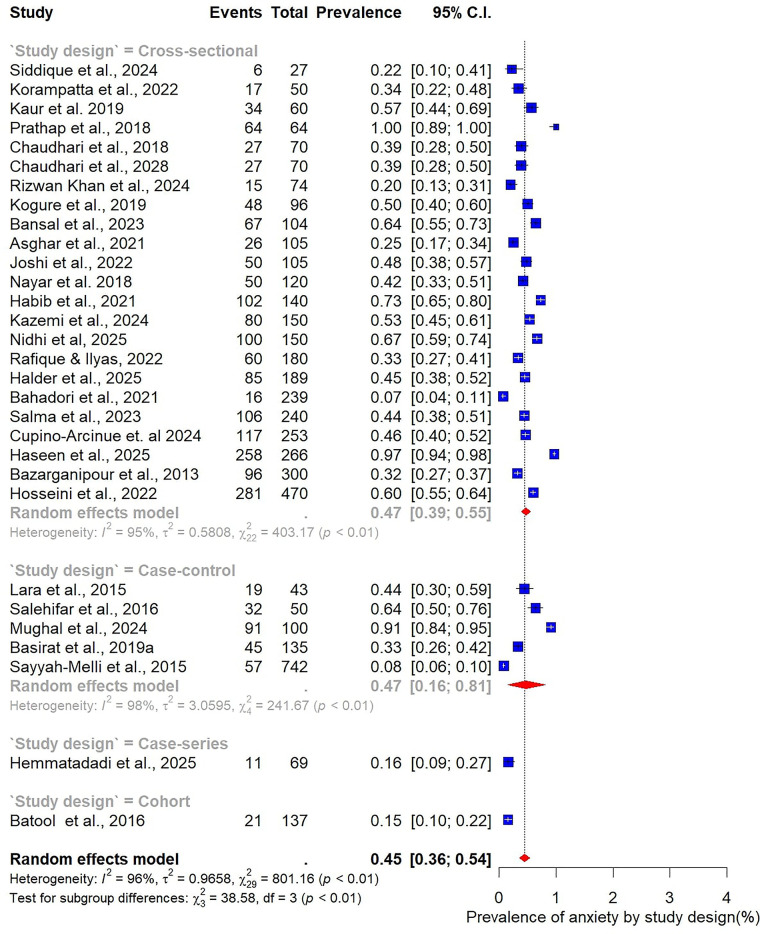
Subgroup analysis of anxiety prevalence in women with PCOS by study design.

**Figure 15:**
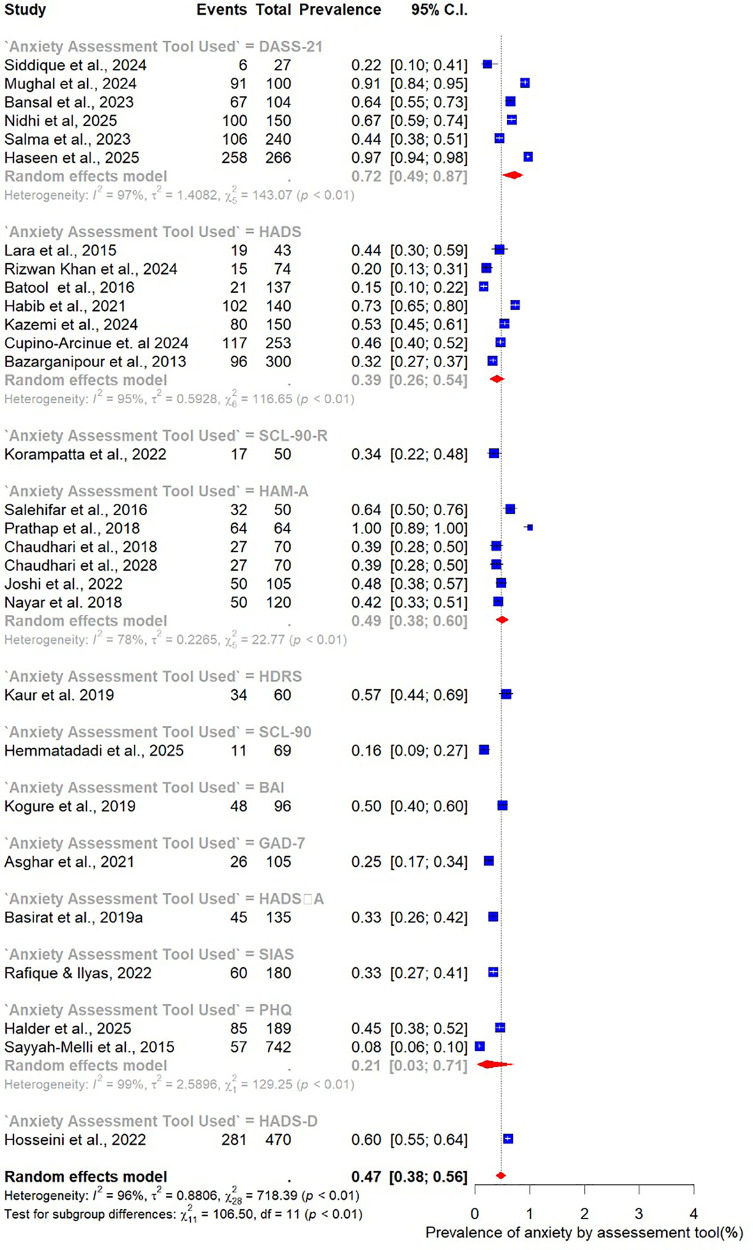
Subgroup analysis of anxiety prevalence in women with PCOS by assessment tool used.

Subgroup analyses were also performed to examine variations in the prevalence of anxiety among women with PCOS, stratified by study and participant characteristics (Figures 10–15).

When stratified by sample size, larger studies with at least 100 participants (*n* = 19) reported a pooled anxiety prevalence of 46% (95% CI: 35–54). This was slightly higher than the pooled prevalence of 41% (95% CI: 30–52) observed in smaller studies with fewer than 100 participants (*n* = 11) (Figure 10).

Analysis by age group revealed that younger women tended to report higher prevalence of anxiety. The highest prevalence was observed among those aged 20–25 years at 56% (95% CI: 32–77; *n* = 5). Women in the 26–30-year age group, which contributed the largest number of studies (*n* = 14), had a prevalence of 49% (95% CI: 39–59). In comparison, the 21–25-year age group reported a prevalence of 46% (95% CI: 16–79; *n* = 5), while the 31–35-year group had the lowest prevalence at 40% (95% CI: 27–53; *n* = 5). Taken together, these results indicate that the burden of anxiety is greatest among women in their early reproductive years, particularly between 20 and 25 years of age (Figure 11).

Differences were also evident when stratified by BMI. Studies that included only normal-weight participants (*n* = 5) reported a pooled anxiety prevalence of 46% (95% CI: 33–59). In contrast, studies focusing on overweight participants (*n* = 11) found a substantially lower prevalence of 32% (95% CI: 20–48) (Figure 12).

The analysis by geographic region highlighted marked variability. Studies from India (*n* = 11) reported the highest pooled prevalence of 51% (95% CI: 43–60), while those from Pakistan (*n* = 7) yielded a prevalence of 40% (95% CI: 20–63). Studies conducted in Iran (*n* = 7) reported the lowest prevalence at 25% (95% CI: 12–46) (Figure 13).

When stratified by study design, the pooled prevalence estimates were strikingly similar across designs. The 23 cohort studies reported a prevalence of 47% (95% CI: 39–55; I² = 95%), while the five case–control studies yielded an identical prevalence of 47% (95% CI: 16–81; I² = 98%). However, the wider confidence intervals observed in case–control studies reflect greater uncertainty and variability in their estimates compared to cohort designs (Figure 14).

Finally, subgroup analysis by assessment tool revealed substantial variation in prevalence estimates depending on the measurement instrument used. The Depression, Anxiety, and Stress Scale (DASS-21; *n* = 7) produced the highest pooled prevalence of anxiety at 72% (95% CI: 49–87). The Hamilton Anxiety Rating Scale (HAM-A; *n* = 7) yielded a prevalence of 49% (95% CI: 38–60), while the Hospital Anxiety and Depression Scale (HADS; *n* = 8) reported the lowest prevalence at 39% (95% CI: 26–54) (Figure 15).

### 3.6 Factors associated with depression and anxiety

In addition to estimating prevalence, this review examined whether common clinical features of PCOS were associated with an increased risk of depression or anxiety (See Supplementary File S7). The pooled analyses focused on infertility, hirsutism, and acne, which are among the most frequently reported and clinically relevant manifestations of PCOS.

For depression, women with infertility problems were found to have 46% higher odds of reporting depressive symptoms compared to those without infertility (pooled OR = 1.46, 95% CI: 0.90–2.38). Similarly, hirsutism was associated with a modestly elevated odds of depression (pooled OR = 1.17, 95% CI: 0.91–1.51). Acne also showed a positive association, with women experiencing acne demonstrating 40% higher odds of depressive symptoms (pooled OR = 1.40, 95% CI: 0.75–2.59). However, in all cases, the confidence intervals crossed unity, indicating that these associations were not statistically significant across the body of evidence.

For anxiety, the patterns were broadly similar. Women with acne had a pooled OR of 1.43 (95% CI: 0.83–2.46), suggesting a potential but non-significant increase in the odds of experiencing anxiety symptoms. Hirsutism also demonstrated a comparable association, with a pooled OR of 1.25 (95% CI: 0.75–2.07). As with depression, these associations did not reach statistical significance, reflecting variability in study findings and limited statistical power in the available data.

### 3.7 Publication bias

An analysis of publication bias using Egger's test and funnel plots indicated no evidence of publication bias in the estimation of pooled prevalence for both depression and anxiety. Although Egger's test and conventional funnel plots showed no evidence of small-study effects, these methods are known to be unreliable for high-heterogeneity proportion meta-analyses. For depression, the overall Egger's test was non-significant (t = 0.96, df = 36, *p* = 0.3444), with similar non-significant results observed in subgroup analyses by sample size: studies with sample size <100 (t = 1.94, df = 12, *p* = 0.0766) and ≥100 (t = 1.13, df = 22, *p* = 0.2721). Likewise, no significant publication bias was detected for the pooled prevalence of anxiety, with Egger's test results of t = 0.31, df = 28, *p* = 0.7558 overall, and non-significant findings in subgroups with sample size <100 (t = 0.33, df = 9, *p* = 0.7489) and ≥100 (t = 0.44, df = 17, *p* = 0.6635). The corresponding funnel plots for both depression and anxiety (See Supplementary File S8) assessments demonstrated symmetrical shapes, further supporting the absence of publication bias, and are provided in the supplementary file. In addition, the trim-and-fill analysis (See Supplementary File S9) suggested no significant publication bias, as the adjusted pooled prevalence of depression and anxiety remained consistent with the original estimate, indicating robustness of the results.

### 3.8 Sensitivity analysis

The leave-one-out sensitivity analysis indicated that the pooled prevalence of depression remained stable, with most individual studies not exerting a significant influence on the overall estimate. In contrast, the analysis for anxiety revealed that although most studies had minimal impact, a few contributed to slight variations from the pooled prevalence of 45%. (See Supplementary File S10).

### 3.9 GRADE assessment

The certainty of evidence for the main outcomes was generally rated as low due to methodological limitations, substantial heterogeneity, and variability across studies. (See Supplementary File S6). For depression prevalence, 38 studies reported a pooled prevalence of 51% (95% CI: 43%–59%), with substantial heterogeneity (I² = 97%). The evidence was graded as low certainty, meaning the true prevalence may differ from the pooled estimate, though the burden of depression in women with PCOS in LMICs is consistently high across studies. For anxiety prevalence, 30 studies yielded a pooled prevalence of 45% (95% CI: 36%–54%), also with substantial heterogeneity (I² = 96%). This outcome was likewise graded as low certainty, reflecting concerns about inconsistency and study quality. Subgroup analyses provided additional insights but were also graded as low certainty. Women aged 20–25 years consistently showed higher rates of depression and anxiety (58%–63%) compared to women aged ≥26 years (49%–51%). Geographically, studies from India reported slightly higher depression prevalence (55%) compared to the overall LMIC average (51%). Analyses of clinical features (infertility, hirsutism, and acne) suggested modestly increased odds of depression and anxiety (OR range: 1.17–1.46), but none of these associations reached statistical significance. These findings were downgraded due to imprecision and methodological limitations.

## 4 Discussion

This systematic review and meta-analysis is, to our knowledge, the first to comprehensively synthesize evidence on the prevalence of depression and anxiety among women of reproductive age with PCOS in LMICs. We found that approximately half of women with PCOS in these settings experienced clinically significant symptoms of depression (51%) and anxiety (45%). These prevalence levels are markedly higher than those observed in global estimates, where depression and anxiety have been reported in 30%–40% of women with PCOS (10, 77–79). This finding suggests that women with PCOS in LMICs face a disproportionate psychological burden, highlighting the interplay between reproductive health disorders and mental health in resource-constrained environments.

Our findings align with prior systematic reviews indicating elevated psychiatric morbidity among women with PCOS worldwide, but the magnitude observed in LMICs appears greater (10). Several factors may explain this disparity. First, limited access to healthcare services and delayed diagnosis in LMICs may exacerbate symptom severity and prolong distress (80, 81). Second, sociocultural pressures surrounding fertility and marriage, particularly acute in South Asian and sub-Saharan African contexts, may intensify the psychosocial impact of PCOS (82, 83). In South Asian contexts, where fertility and motherhood are strongly tied to women's social identity and marital stability, those with infertility or delayed conception often face marital pressure and social stigma (84–86). Similar patterns of psychosocial distress have been reported globally, where visible symptoms such as hirsutism, acne, and obesity may provoke negative body image, social withdrawal, and reduced quality of life. Evidence from systematic review demonstrates that body image dissatisfaction, perceived stigma, and low self-esteem mediate the relationship between PCOS and adverse mental health outcomes, particularly depression and anxiety (11). These cultural and appearance-related pressures contribute to internalized shame and vulnerability to psychological distress among women with PCOS. Third, stigma associated with both mental health disorders and reproductive conditions can compound distress and discourage help-seeking behaviors. These contextual stressors likely contribute to the higher prevalence observed in our review compared to studies from high-income countries.

There was considerable heterogeneity among studies, which is also comparable with previous meta-analyses conducted among PCOS populations (10, 83). The inconsistency was probably caused by variability in the PCOS diagnostic criteria, the sample sizes, and the application of various screening tools. As an example, methods that employed the BDI or the DASS-21 showed higher prevalence estimates as compared to those that employed the HADS due to differences in methods of sensitivity and coverage of symptoms (87). In addition to these methodological considerations, there are a number of possible sources of bias that may have contributed to the pooled estimates. A high percentage of the studies were clinical, which can increase the prevalence since the symptomatic women tend to seek care more. Non-equivalence of measurements in studies because of the different instruments and locally invalid cut-offs may have contributed to differing case classification. Observed prevalence may also be influenced by cultural differences in the manifestation and reporting of psychological distress, in which cases, somatic symptoms or the underreporting of stigma may occur. Moreover, there could also be selection bias due to convenience sampling and lack of representativeness of study populations as another factor that could have increased heterogeneity. Although this variability restricts the accuracy of pooled estimates, it highlights the fact that mental health research on PCOS needs standardized diagnostic and assessment protocols and that the need is now more than ever in LMICs.

Our subgroup findings provide additional insights into vulnerable groups. Younger women, especially those in their early twenties, appeared to be disproportionately affected by both depression and anxiety. This is consistent with prior research indicating that younger women with PCOS face heightened psychological strain due to body image concerns, menstrual irregularities, and anxiety surrounding fertility (88, 89). Other differences were also geographical, as it was found to be more prevalent in India and Pakistan than in Iran, which can be attributed to cultural and societal factors. The issue of reproductive health and menstrual issues tends to be highly connected with the notions of femininity, fertility, and marriage appropriateness in South Asian contexts, which are strong aspects of sociocultural and family organization (15). The women affected with PCOS might consequently encounter more psychosocial distress because of community-based stigma over infertility, hirsutism, and body image issues, which is likely to be regarded morally or aesthetically, and not biomedically (52, 90). These patterns reinforce the importance of contextual and cultural factors in shaping psychological outcomes among women with PCOS.

Although infertility, hirsutism, and acne were associated with increased odds of depression and anxiety, these associations did not reach statistical significance in pooled analyses. Nonetheless, the direction of effect aligns with prior evidence showing that dermatological and reproductive manifestations of PCOS can lead to perceived stigma, reduced self-esteem, and poorer quality of life (11, 12, 15, 79). The stigma associated with PCOS arises largely from its visible and reproductive symptoms including hirsutism, acne, obesity, and infertility which contradict cultural ideals of femininity and fertility in many LMIC settings. These perceptions can result in social judgment, marital pressure, and internalized shame, all of which contribute to depression and anxiety. This pattern mirrors findings from other contexts, such as the COVID-19 pandemic, where public stigma was shown to elevate depression risk (91).

Publication bias was not evident which supported the robustness of the findings. However, several limitations must be acknowledged. The cross-sectional nature of most included studies precludes causal inference about the relationship between PCOS features and mental health outcomes. Moreover, the lack of uniform diagnostic criteria for PCOS and the variability in study settings may limit comparability across studies. Overall, while the pooled prevalence estimates indicate a substantial psychological burden in women with PCOS in LMICs, the low certainty of evidence underscores the need for higher-quality, standardized studies to strengthen future estimates.

The implications of these findings are substantial. First, they highlight the need to integrate routine mental health screening into reproductive and endocrine clinics, particularly in LMICs where PCOS is underdiagnosed and mental health services are scarce. Second, culturally tailored interventions that address stigma, fertility concerns, and body image should be prioritized to improve psychosocial outcomes for women with PCOS. Third, future research should employ longitudinal designs and standardized diagnostic tools to clarify causal relationships and develop effective interventions. Finally, policymakers and health systems in LMICs must recognize PCOS not only as a reproductive disorder but also as a condition with significant mental health consequences requiring comprehensive and multidisciplinary care.

In conclusion, this review demonstrates that depression and anxiety are highly prevalent among women of reproductive age with PCOS in LMICs, at levels exceeding global averages. The findings underscore the urgent need for context-specific, integrated approaches that address both the physical and psychological dimensions of PCOS. Addressing these unmet needs has the potential to improve quality of life, reproductive health outcomes, and mental wellbeing for millions of women worldwide.

## Data Availability

The original contributions presented in the study are included in the article/Supplementary Material, further inquiries can be directed to the corresponding author.
