# Supplementary material for: Depression and anxiety among women with polycystic ovarian syndrome in low- and middle-income countries: a systematic review and meta-analysis
Source: Front Glob Womens Health. 2025 Nov 25;6:1688913. doi: 10.3389/fgwh.2025.1688913 (PMC12685914; doi:10.3389/fgwh.2025.1688913)
Supplement: Supplementary file 2 [file Presentation1.pdf]

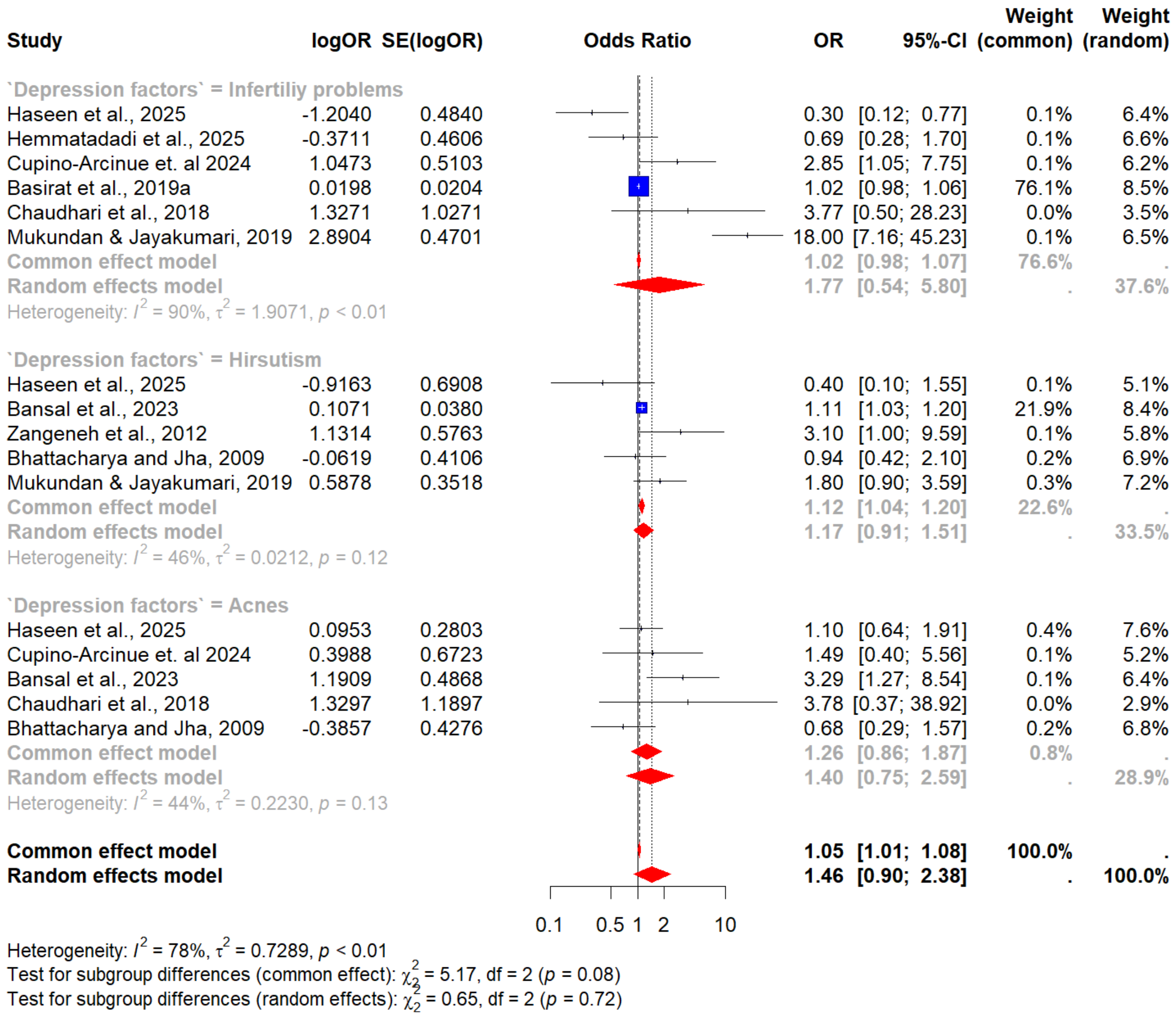

Figure 1: Forest Plot of Odds Ratios for factors associated with Depression in PCOS patients

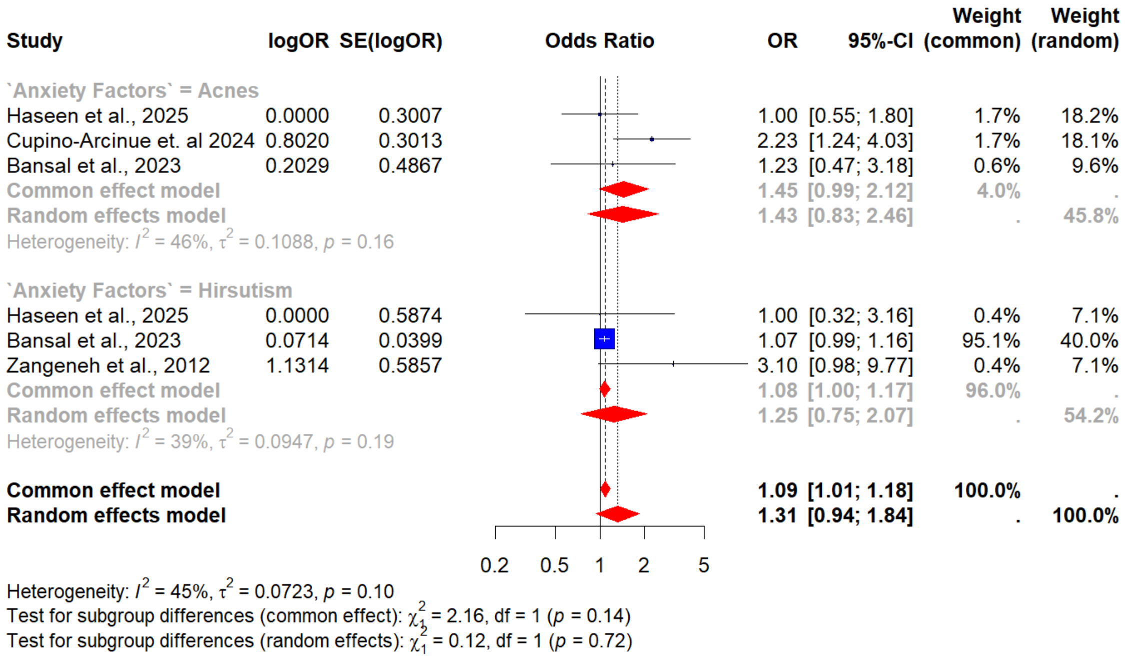

Figure 2: Forest Plot of Odds Ratios for factors associated with Anxiety in PCOS patients
