# Supplementary material for: Depression and anxiety among women with polycystic ovarian syndrome in low- and middle-income countries: a systematic review and meta-analysis
Source: Front Glob Womens Health. 2025 Nov 25;6:1688913. doi: 10.3389/fgwh.2025.1688913 (PMC12685914; doi:10.3389/fgwh.2025.1688913)
Supplement: Supplementary file 3 [file Presentation2.pdf]

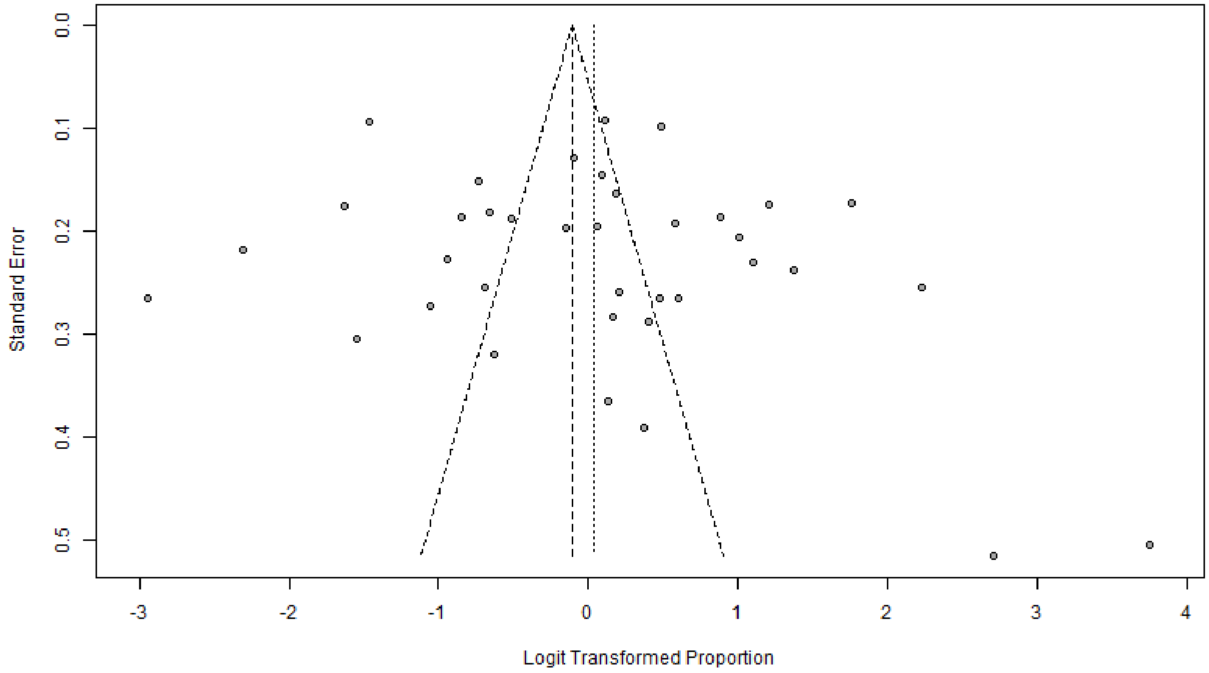

Figure 1: Funnel Plot of meta-analysis of Depression in Patients with PCOS

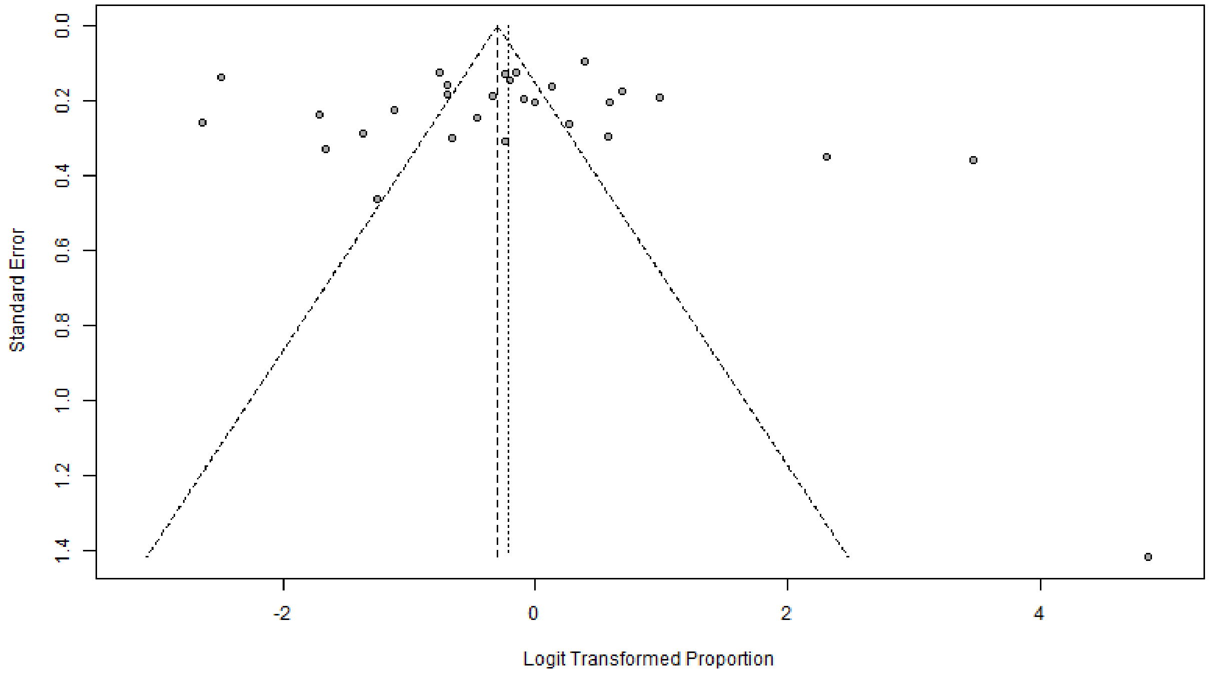

Figure 2: Funnel Plot of meta-analysis of Anxiety in Patients with PCOS
