# Supplementary material for: Depression and anxiety among women with polycystic ovarian syndrome in low- and middle-income countries: a systematic review and meta-analysis
Source: Front Glob Womens Health. 2025 Nov 25;6:1688913. doi: 10.3389/fgwh.2025.1688913 (PMC12685914; doi:10.3389/fgwh.2025.1688913)
Supplement: Supplementary file 4 [file Presentation3.pdf]

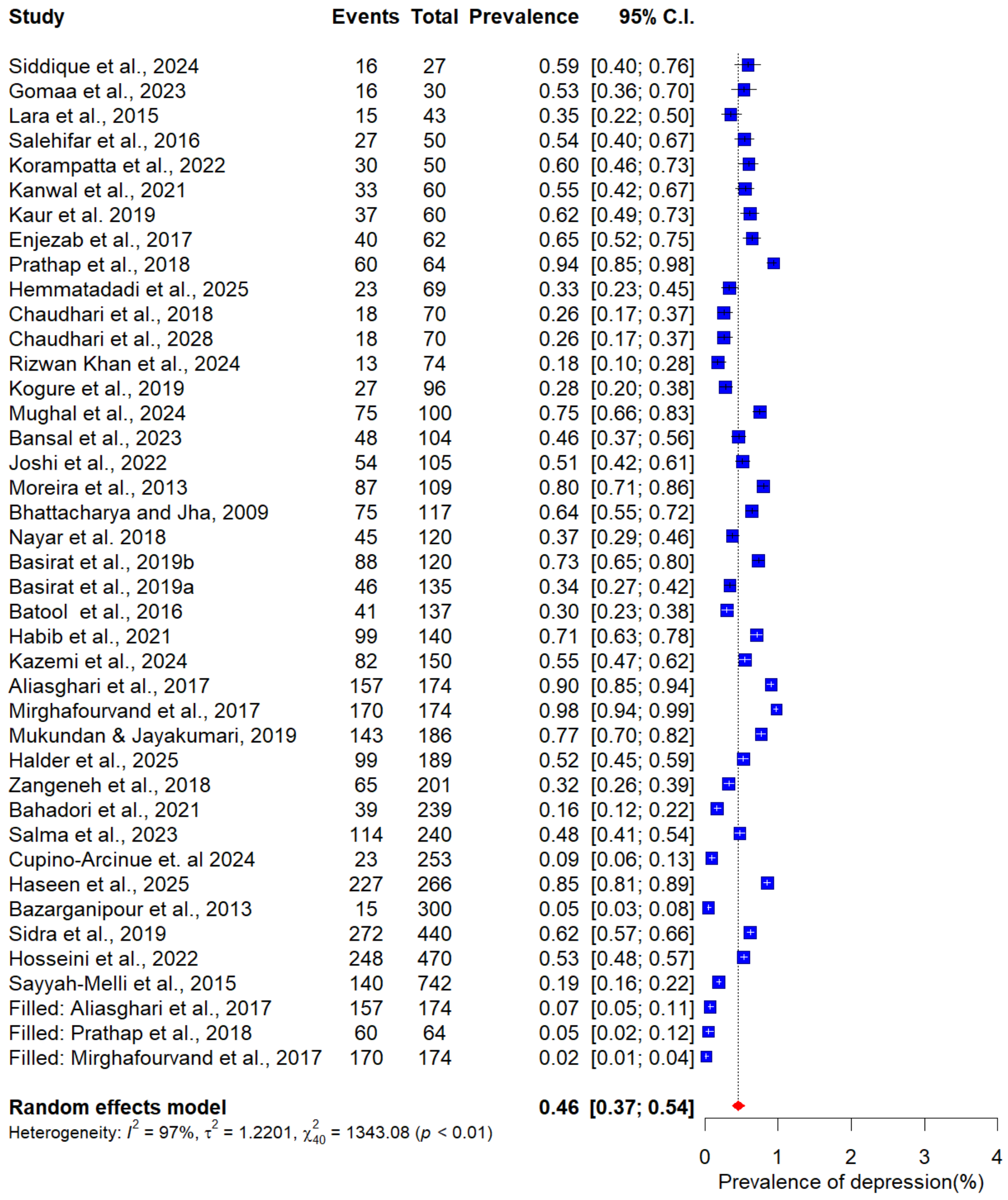

Figure 1: Trim and fill analysis of meta-analysis of Depression in Patients with PCOS

Study	Events	Total	Prevalence	95% C.I.
Siddique et al., 2024	6	27	0.22	[0.10; 0.41]
Hemmatadadi et al., 2025	11	69	0.16	[0.09; 0.27]
Rizwan Khan et al., 2024	15	74	0.20	[0.13; 0.31]
Bahadori et al., 2021	16	239	0.07	[0.04; 0.11]
Korampatta et al., 2022	17	50	0.34	[0.22; 0.48]
Lara et al., 2015	19	43	0.44	[0.30; 0.59]
Batool et al., 2016	21	137	0.15	[0.10; 0.22]
Asghar et al., 2021	26	105	0.25	[0.17; 0.34]
Chaudhari et al., 2018	27	70	0.39	[0.28; 0.50]
Chaudhari et al., 2028	27	70	0.39	[0.28; 0.50]
Salehifar et al., 2016	32	50	0.64	[0.50; 0.76]
Kaur et al. 2019	34	60	0.57	[0.44; 0.69]
Basirat et al., 2019a	45	135	0.33	[0.26; 0.42]
Kogure et al., 2019	48	96	0.50	[0.40; 0.60]
Joshi et al., 2022	50	105	0.48	[0.38; 0.57]
Nayar et al. 2018	50	120	0.42	[0.33; 0.51]
Sayyah-Melli et al., 2015	57	742	0.08	[0.06; 0.10]
Rafique & Ilyas, 2022	60	180	0.33	[0.27; 0.41]
Prathap et al., 2018	64	64	0.99	[0.89; 1.00]
Bansal et al., 2023	67	104	0.64	[0.55; 0.73]
Kazemi et al., 2024	80	150	0.53	[0.45; 0.61]
Halder et al., 2025	85	189	0.45	[0.38; 0.52]
Mughal et al., 2024	91	100	0.91	[0.84; 0.95]
Bazarganipour et al., 2013	96	300	0.32	[0.27; 0.37]
Nidhi et al, 2025	100	150	0.67	[0.59; 0.74]
Habib et al., 2021	102	140	0.73	[0.65; 0.80]
Salma et al., 2023	106	240	0.44	[0.38; 0.51]
Cupino-Arcinue et. al 2024	117	253	0.46	[0.40; 0.52]
Haseen et al., 2025	258	266	0.97	[0.94; 0.98]
Hosseini et al., 2022	281	470	0.60	[0.55; 0.64]
Filled: Prathap et al., 2018	64	64	0.00	[0.00; 0.06]
**Random effects model**			**0.43**	**[0.34; 0.52]**

Heterogeneity: $I^2 = 96\%$, $\tau^2 = 0.9804$, $\chi^2_{30} = 814.40$ ($p < 0.01$)

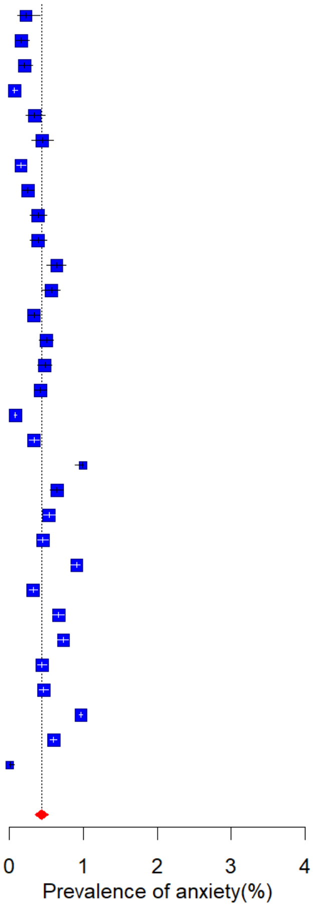

Figure 2: Trim and fill analysis of meta-analysis of anxiety in Patients with PCOS
