# Supplementary material for: Depression and anxiety among women with polycystic ovarian syndrome in low- and middle-income countries: a systematic review and meta-analysis
Source: Front Glob Womens Health. 2025 Nov 25;6:1688913. doi: 10.3389/fgwh.2025.1688913 (PMC12685914; doi:10.3389/fgwh.2025.1688913)
Supplement: Supplementary file 8 [file Table3.docx]

**Table 2: Summary of GRADE Assessment Findings**

| **Outcome** | **No. of Studies** | **Effect Estimate (95% CI)** | **Heterogeneity (I²)** | **Certainty of Evidence (GRADE)** |
| --- | --- | --- | --- | --- |
| Depression prevalence | 38 studies | 51% (43–59%) | I² = 97% | ⭑⭑◯◯ Low* |
| Anxiety prevalence | 30 studies | 45% (36–54%) | I² = 96% | ⭑⭑◯◯ Low* |
| Age effect (20–25 yrs vs. ≥26 yrs) | 11 studies | Higher prevalence in younger women (58–63% vs. 49–51%) | High | ⭑⭑◯◯ Low |
| Geographic variation (India vs. others) | 12 studies | India: 55% vs. pooled LMIC average: 51% | High | ⭑⭑◯◯ Low |
| Associated clinical features (infertility, hirsutism, acne) | 6–8 studies each | OR range 1.17–1.46 (not statistically significant) | Moderate | ⭑⭑◯◯ Low |
