# Supplementary material for: Depression and anxiety among women with polycystic ovarian syndrome in low- and middle-income countries: a systematic review and meta-analysis
Source: Front Glob Womens Health. 2025 Nov 25;6:1688913. doi: 10.3389/fgwh.2025.1688913 (PMC12685914; doi:10.3389/fgwh.2025.1688913)
Supplement: Supplementary file 9 [file Table4.docx]

**SEARCH TERMS**

Topic: **Depression and Anxiety among Women of Reproductive Age with Polycystic Ovary Syndrome in LMIC: A Systematic Review and Meta-analysis.**

**Review objectives**

**Primary Review Question**

What is the prevalence of depression and anxiety among women of reproductive age with polycystic ovary syndrome (PCOS)?

**Secondary Review Questions**

How do factors such as demographic, socio-cultural, life-style and economic affect depression and anxiety in women with PCOS?

1. **Keywords
   Depression
   Anxiety**

**Polycystic ovarian Syndrome**

1. **SEARCH TERMS (MeSH Terms)
    polycystic ovarian syndrome:**

“Polycystic ovarian syndrome” OR “Polycystic ovary syndrome” OR PCOS OR “ovary syndrome” OR “Polycystic ovary” OR “Stein-Leventhal syndrome” OR “Sclerocystic Ovarian Degeneration” OR “Ovarian Degeneration” OR “Sclerocystic Ovary Syndrome” OR “polycystic ovarian disease” OR “polycystic ovary” OR “poly-cystic ovary” OR “Ovarian dysfunction”

**AND**

**Depression OR Anxiety:**
depress* OR depression OR “depressive disorders” OR “psychological distress” OR “Mood disorders” OR “depressive symptoms” OR “Major depressive disorder” OR Anxie* OR Anxiety OR “anxiety disorders” OR “Social Anxiety” OR Nervousness OR Hypervigilance OR Anxiousness OR anxious OR “**panic disorder” OR “Social Anxiety Disorder”**

**PUBMED (11-06-2025)**

**SEARCH STRATEGY**

| s/n | Keywords | Search Strategy | Remarks |
| --- | --- | --- | --- |
| #1 | **Depression** | depress*[Title/Abstract] OR depression[Title/Abstract] OR "depressive disorders"[Title/Abstract] OR "psychological distress"[Title/Abstract] OR "Mood disorders"[Title/Abstract] OR "depressive symptoms"[Title/Abstract] OR "Major depressive disorder"[Title/Abstract] | [666,097](https://pubmed.ncbi.nlm.nih.gov/?term=depress%2A%5BTitle%2FAbstract%5D+OR+depression%5BTitle%2FAbstract%5D+OR+%22depressive+disorders%22%5BTitle%2FAbstract%5D+OR+%22psychological+distress%22%5BTitle%2FAbstract%5D+OR+%22Mood+disorders%22%5BTitle%2FAbstract%5D+OR+%22depressive+symptoms%22%5BTitle%2FAbstract%5D+OR+%22Major+depressive+disorder%22%5BTitle%2FAbstract%5D&sort=) |
| #2 | **Anxiety** | Anxie*[Title/Abstract] OR Anxiety[Title/Abstract] OR "anxiety disorders"[Title/Abstract] OR "Social Anxiety"[Title/Abstract] OR Nervousness[Title/Abstract] OR Hypervigilance[Title/Abstract] OR Anxiousness[Title/Abstract] OR anxious[Title/Abstract] OR "panic disorder"[Title/Abstract] OR "Social Anxiety Disorder"[Title/Abstract] | [329,760](https://pubmed.ncbi.nlm.nih.gov/?term=Anxie%2A%5BTitle%2FAbstract%5D+OR+Anxiety%5BTitle%2FAbstract%5D+OR+%22anxiety+disorders%22%5BTitle%2FAbstract%5D+OR+%22Social+Anxiety%22%5BTitle%2FAbstract%5D+OR+Nervousness%5BTitle%2FAbstract%5D+OR+Hypervigilance%5BTitle%2FAbstract%5D+OR+Anxiousness%5BTitle%2FAbstract%5D+OR+anxious%5BTitle%2FAbstract%5D+OR+%22panic+disorder%22%5BTitle%2FAbstract%5D+OR+%22Social+Anxiety+Disorder%22%5BTitle%2FAbstract%5D&ac=no&sort=relevance) |
| #3 | **(#1 OR #2)** | "depress*"[Title/Abstract] OR "depression"[Title/Abstract] OR "depressive disorders"[Title/Abstract] OR "psychological distress"[Title/Abstract] OR "Mood disorders"[Title/Abstract] OR "depressive symptoms"[Title/Abstract] OR "Major depressive disorder"[Title/Abstract] OR "anxie*"[Title/Abstract] OR "Anxiety"[Title/Abstract] OR "anxiety disorders"[Title/Abstract] OR "Social Anxiety"[Title/Abstract] OR "Nervousness"[Title/Abstract] OR "Hypervigilance"[Title/Abstract] OR "Anxiousness"[Title/Abstract] OR "anxious"[Title/Abstract] OR "panic disorder"[Title/Abstract] OR "Social Anxiety Disorder"[Title/Abstract] | [822,319](https://pubmed.ncbi.nlm.nih.gov/?term=%28%231+OR+%232%29&ac=no&sort=relevance) |
| #4 | **Polycystic ovarian Syndrome** | "Polycystic ovarian syndrome"[Title/Abstract] OR "Polycystic ovary syndrome"[Title/Abstract] OR PCOS[Title/Abstract] OR "ovary syndrome"[Title/Abstract] OR "Polycystic ovary"[Title/Abstract] OR "Stein-Leventhal syndrome"[Title/Abstract] OR "Sclerocystic Ovarian Degeneration"[Title/Abstract] OR "Ovarian Degeneration"[Title/Abstract] OR "Sclerocystic Ovary Syndrome"[Title/Abstract] OR "polycystic ovarian disease"[Title/Abstract] OR "polycystic ovary"[Title/Abstract] OR "poly-cystic ovary"[Title/Abstract] OR "Ovarian dysfunction"[Title/Abstract] | [27,251](https://pubmed.ncbi.nlm.nih.gov/?term=%22Polycystic+ovarian+syndrome%22%5BTitle%2FAbstract%5D+OR+%22Polycystic+ovary+syndrome%22%5BTitle%2FAbstract%5D+OR+PCOS%5BTitle%2FAbstract%5D+OR+%22ovary+syndrome%22%5BTitle%2FAbstract%5D+OR+%22Polycystic+ovary%22%5BTitle%2FAbstract%5D+OR+%22Stein-Leventhal+syndrome%22%5BTitle%2FAbstract%5D+OR+%22Sclerocystic+Ovarian+Degeneration%22%5BTitle%2FAbstract%5D+OR+%22Ovarian+Degeneration%22%5BTitle%2FAbstract%5D+OR+%22Sclerocystic+Ovary+Syndrome%22%5BTitle%2FAbstract%5D+OR+%22polycystic+ovarian+disease%22%5BTitle%2FAbstract%5D+OR+%22polycystic+ovary%22%5BTitle%2FAbstract%5D+OR+%22poly-cystic+ovary%22%5BTitle%2FAbstract%5D+OR+%22Ovarian+dysfunction%22%5BTitle%2FAbstract%5D&ac=no&sort=relevance) |
| #5 | **#3 AND #4** | ("depress*"[Title/Abstract] OR "depression"[Title/Abstract] OR "depressive disorders"[Title/Abstract] OR "psychological distress"[Title/Abstract] OR "Mood disorders"[Title/Abstract] OR "depressive symptoms"[Title/Abstract] OR "Major depressive disorder"[Title/Abstract] OR ("anxie*"[Title/Abstract] OR "Anxiety"[Title/Abstract] OR "anxiety disorders"[Title/Abstract] OR "Social Anxiety"[Title/Abstract] OR "Nervousness"[Title/Abstract] OR "Hypervigilance"[Title/Abstract] OR "Anxiousness"[Title/Abstract] OR "anxious"[Title/Abstract] OR "panic disorder"[Title/Abstract] OR "Social Anxiety Disorder"[Title/Abstract])) AND ("Polycystic ovarian syndrome"[Title/Abstract] OR "Polycystic ovary syndrome"[Title/Abstract] OR "PCOS"[Title/Abstract] OR "ovary syndrome"[Title/Abstract] OR "Polycystic ovary"[Title/Abstract] OR "Stein-Leventhal syndrome"[Title/Abstract] OR "Sclerocystic Ovarian Degeneration"[Title/Abstract] OR "Ovarian Degeneration"[Title/Abstract] OR "Sclerocystic Ovary Syndrome"[Title/Abstract] OR "polycystic ovarian disease"[Title/Abstract] OR "Polycystic ovary"[Title/Abstract] OR "poly-cystic ovary"[Title/Abstract] OR "Ovarian dysfunction"[Title/Abstract]) | [927](https://pubmed.ncbi.nlm.nih.gov/?term=%233+AND+%234&ac=no&sort=relevance) |
| #6 | **#3 AND #4 Filters: from 2005/1/1 - 2025/6/13** | (("depress*"[Title/Abstract] OR "depression"[Title/Abstract] OR "depressive disorders"[Title/Abstract] OR "psychological distress"[Title/Abstract] OR "Mood disorders"[Title/Abstract] OR "depressive symptoms"[Title/Abstract] OR "Major depressive disorder"[Title/Abstract] OR ("anxie*"[Title/Abstract] OR "Anxiety"[Title/Abstract] OR "anxiety disorders"[Title/Abstract] OR "Social Anxiety"[Title/Abstract] OR "Nervousness"[Title/Abstract] OR "Hypervigilance"[Title/Abstract] OR "Anxiousness"[Title/Abstract] OR "anxious"[Title/Abstract] OR "panic disorder"[Title/Abstract] OR "Social Anxiety Disorder"[Title/Abstract])) AND ("Polycystic ovarian syndrome"[Title/Abstract] OR "Polycystic ovary syndrome"[Title/Abstract] OR "PCOS"[Title/Abstract] OR "ovary syndrome"[Title/Abstract] OR "Polycystic ovary"[Title/Abstract] OR "Stein-Leventhal syndrome"[Title/Abstract] OR "Sclerocystic Ovarian Degeneration"[Title/Abstract] OR "Ovarian Degeneration"[Title/Abstract] OR "Sclerocystic Ovary Syndrome"[Title/Abstract] OR "polycystic ovarian disease"[Title/Abstract] OR "Polycystic ovary"[Title/Abstract] OR "poly-cystic ovary"[Title/Abstract] OR "Ovarian dysfunction"[Title/Abstract])) AND (2005/1/1:2025/6/13[pdat]) | [861](https://pubmed.ncbi.nlm.nih.gov/?term=%233+AND+%234&filter=dates.2005%2F1%2F1-2025%2F6%2F13&ac=no&sort=relevance) |

**CIHNYL (16-06-2025)**

XB (depress* OR depression OR “depressive disorders” OR “psychological distress” OR “Mood disorders” OR “depressive symptoms” OR “Major depressive disorder” OR Anxie* OR Anxiety OR “anxiety disorders” OR “Social Anxiety” OR Nervousness OR Hypervigilance OR Anxiousness OR anxious OR “panic disorder” OR “Social Anxiety Disorder”) AND XB (“Polycystic ovarian syndrome” OR “Polycystic ovary syndrome” OR PCOS OR “ovary syndrome” OR “Polycystic ovary” OR “Stein-Leventhal syndrome” OR “Sclerocystic Ovarian Degeneration” OR “Ovarian Degeneration” OR “Sclerocystic Ovary Syndrome” OR “polycystic ovarian disease” OR “polycystic ovary” OR “poly-cystic ovary” OR “Ovarian dysfunction”) **272**

**SCOPUS (16-06-2025)**

( TITLE-ABS-KEY ( depress* OR depression OR "depressive disorders" OR "psychological distress" OR "Mood disorders" OR "depressive symptoms" OR "Major depressive disorder" OR anxie* OR anxiety OR "anxiety disorders" OR "Social Anxiety" OR nervousness OR hypervigilance OR anxiousness OR anxious OR "panic disorder" OR "Social Anxiety Disorder" ) AND TITLE-ABS-KEY ( "Polycystic ovarian syndrome" OR "Polycystic ovary syndrome" OR pcos OR "ovary syndrome" OR "Polycystic ovary" OR "Stein-Leventhal syndrome" OR "Sclerocystic Ovarian Degeneration" OR "Ovarian Degeneration" OR "Sclerocystic Ovary Syndrome" OR "polycystic ovarian disease" OR "polycystic ovary" OR "poly-cystic ovary" OR "Ovarian dysfunction" ) ) AND PUBYEAR > 2004 **1455**

**WEB OF SCIENCE SEARCH STRATEGY (16-06-2025)**

| s/n | Keywords | Search Strategy | Remarks |
| --- | --- | --- | --- |
| #1 | **Depression OR Anxiety** | TS=(depress* OR depression OR “depressive disorders” OR “psychological distress” OR “Mood disorders” OR “depressive symptoms” OR “Major depressive disorder” OR Anxie* OR Anxiety OR “anxiety disorders” OR “Social Anxiety” OR Nervousness OR Hypervigilance OR Anxiousness OR anxious OR “panic disorder” OR “Social Anxiety Disorder”) and Preprint Citation Index (Exclude – Database) | [1,960,911](https://www-webofscience-com.proxy.kib.ki.se/wos/alldb/summary/9119aec3-232d-4162-97fe-14329d6bd174-01689c5aae/relevance/1) |
| #2 | **Polycystic ovarian Syndrome** | TS=(“Polycystic ovarian syndrome” OR “Polycystic ovary syndrome” OR PCOS OR “ovary syndrome” OR “Polycystic ovary” OR “Stein-Leventhal syndrome” OR “Sclerocystic Ovarian Degeneration” OR “Ovarian Degeneration” OR “Sclerocystic Ovary Syndrome” OR “polycystic ovarian disease” OR “polycystic ovary” OR “poly-cystic ovary” OR “Ovarian dysfunction”) and Preprint Citation Index (Exclude – Database) | [48,216](https://www-webofscience-com.proxy.kib.ki.se/wos/alldb/summary/4e42eca6-58f7-4a10-9c91-9b17bd03b732-01689c65bf/relevance/1) |
| #3 | **#1 AND #2** | #1 AND #2 and Preprint Citation Index (Exclude – Database) | [1,832](https://www-webofscience-com.proxy.kib.ki.se/wos/alldb/summary/78e4dc14-a8be-4c9f-a0ee-d45672cf0518-01689c6775/relevance/1) |
| #4 | **#3  Filters: from 2005/1/1 - 2025/6/15** | #1 AND #2 and Preprint Citation Index (Exclude – Database) and 2025 or 2024 or 2023 or 2022 or 2021 or 2020 or 2019 or 2018 or 2017 or 2016 or 2014 or 2015 or 2013 or 2012 or 2011 or 2009 or 2010 or 2008 or 2007 or 2006 or 2005 (Publication Years) | [1,681](https://www-webofscience-com.proxy.kib.ki.se/wos/alldb/summary/4eb404f8-3528-4325-b12e-99cfb05e8ddd-01689c6aa1/relevance/1) |
| #5 | **Filters** | #1 AND #2 and Preprint Citation Index (Exclude – Database) and 2025 or 2024 or 2023 or 2022 or 2021 or 2020 or 2019 or 2018 or 2017 or 2016 or 2014 or 2015 or 2013 or 2012 or 2011 or 2009 or 2010 or 2008 or 2007 or 2006 or 2005 (Publication Years) and Article (Document Types) and Web of Science Core Collection (Database) | [1,268](https://www-webofscience-com.proxy.kib.ki.se/wos/alldb/summary/cc74a080-5f92-4e3e-ac3a-54b6fdc3e104-01689c6db3/relevance/1) |
