# Supplementary material for: Depression and anxiety among women with polycystic ovarian syndrome in low- and middle-income countries: a systematic review and meta-analysis
Source: Front Glob Womens Health. 2025 Nov 25;6:1688913. doi: 10.3389/fgwh.2025.1688913 (PMC12685914; doi:10.3389/fgwh.2025.1688913)
Supplement: Supplementary file 10 [file Table5.docx]

**QUALITY ASSESSMENT TEMPLATE**

| **Article no.** | **Study** | **Q1** | **Q2** | **Q3** | **Q4** | **Q5** | **Q6** | **Q7** | **Q8** | **Total score** | **Quality**  **score (%)** | **Risk of Bias** |
| --- | --- | --- | --- | --- | --- | --- | --- | --- | --- | --- | --- | --- |
|  | Aliasghari et al. (29) | 1 | 1 | 1 | 1 | 0 | 0 | 1 | 1 | 6 | 75 | **Low** |
|  | Asghar et al. (30) | 1 | 1 | 1 | 1 | 1 | 0 | 1 | 0 | 6 | 75 | **Low** |
|  | Bahadori et al. (31) | 1 | 1 | 1 | 1 | 0 | 0 | 1 | 1 | 6 | 75 | **Low** |
|  | Bansal et al. (32) | 1 | 1 | 1 | 1 | 1 | 1 | 1 | 1 | 8 | 100 | **Low** |
|  | Basirat et al. (34) | 1 | 1 | 1 | 1 | 1 | 0 | 1 | 0 | 6 | 75 | **Low** |
|  | Basirat et al. (33) | 1 | 1 | 1 | 1 | 0 | 0 | 1 | 1 | 6 | 75 | **Low** |
|  | Batool et al., (35) | 1 | 1 | 1 | 1 | 1 | 1 | 1 | 1 | 8 | 100 | **Low** |
|  | Bazarganipour et al. (36) | 1 | 1 | 1 | 1 | 1 | 0 | 1 | 0 | 6 | 75 | **Low** |
|  | Bhattacharya and Jha,(37) | 1 | 1 | 1 | 1 | 1 | 1 | 1 | 1 | 8 | 100 | **Low** |
|  | Chaudhari et al. (38) | 1 | 1 | 1 | 1 | 0 | 1 | 1 | 1 | 7 | 87.5 | **Low** |
|  | Cupino-Arcinue et. al (39) | 1 | 1 | 1 | 1 | 1 | 1 | 1 | 1 | 8 | 100 | **Low** |
|  | Enjezab et al. (40) | 1 | 1 | 1 | 1 | 1 | 0 | 1 | 0 | 6 | 75 | **Low** |
|  | Gomaa et al. (41) | 1 | 1 | 1 | 1 | 1 | 0 | 1 | 1 | 7 | 87.5 | **Low** |
|  | Habib et al. (42) | 1 | 1 | 1 | 1 | 0 | 0 | 1 | 1 | 6 | 75 | **Low** |
|  | Halder et al. (43) | 1 | 1 | 1 | 1 | 1 | 1 | 1 | 1 | 8 | 100 | **Low** |
|  | Haseen et al. (44) | 1 | 1 | 1 | 1 | 1 | 0 | 1 | 1 | 7 | 87.5 | **Low** |
|  | Hemmatadadi et al. (45) | 1 | 1 | 1 | 1 | 1 | 0 | 1 | 1 | 6 | 87.5 | **Low** |
|  | Hosseini et al. (68) | 1 | 1 | 1 | 1 | 0 | 0 | 1 | 1 | 6 | 75 | **Low** |
|  | Joshi et al., (46) | 1 | 1 | 1 | 1 | 0 | 0 | 1 | 1 | 6 | 75 | **Low** |
|  | Kanwal et al. (47) |  |  |  |  |  |  |  |  | 7 | 87.5 | **Low** |
|  | Kaur et al. (13) | 1 | 1 | 1 | 1 | 1 | 1 | 1 | 1 | 8 | 100 | **Low** |
|  | Kazemi et al. (72) | 1 | 1 | 1 | 1 | 1 | 1 | 1 | 1 | 8 | 100 | **Low** |
|  | Kogure et al. (49) | 1 | 1 | 1 | 1 | 1 | 0 | 1 | 1 | 7 | 87.5 | **Low** |
|  | Korampatta et al. (50) | 1 | 1 | 1 | 1 | 1 | 0 | 1 | 0 | 6 | 75 | **Low** |
|  | Lara et al. (51) | 1 | 1 | 1 | 1 | 1 | 0 | 1 | 1 | 7 | 87.5 | **Low** |
|  | Mirghafourvand et al.(52) | 1 | 1 | 1 | 1 | 1 | 1 | 1 | 1 | 7 | 100 | **Low** |
|  | Moreira et al. (53) | 1 | 1 | 1 | 1 | 1 | 1 | 1 | 1 | 8 | 100 | **Low** |
|  | Mughal et al. (54) | 1 | 1 | 1 | 1 | 1 | 0 | 1 | 1 | 7 | 87.5 | **Low** |
|  | Mukundan & Jayakumari, (55) | 1 | 1 | 1 | 1 | 1 | 0 | 1 | 1 | 7 | 87.5 | **Low** |
|  | Nayar et al. (56) | 1 | 1 | 1 | 1 | 1 | 1 | 1 | 1 | 8 | 100 | **Low** |
|  | Nidhi et al. (59) | 1 | 1 | 1 | 1 | 1 | 1 | 1 | 1 | 8 | 100 | **Low** |
|  | Prathap et al. (58) | 1 | 1 | 1 | 1 | 0 | 0 | 1 | 1 | 6 | 75 | **Low** |
|  | Rafique & Ilyas, (60) | 1 | 1 | 1 | 1 | 0 | 0 | 1 | 1 | 6 | 75 | **Low** |
|  | Rizwan Khan et al.(61) | 1 | 1 | 1 | 1 | 1 | 0 | 1 | 0 | 6 | 75 | **Low** |
|  | Salehifar et al. (62) | 1 | 1 | 1 | 1 | 0 | 0 | 1 | 1 | 6 | 75 | **Low** |
|  | Salma et al. (63) | 1 | 1 | 1 | 1 | 1 | 0 | 1 | 1 | 7 | 87.5 | **Low** |
|  | Sayyah-Melli et al. (64) | 1 | 1 | 1 | 1 | 1 | 0 | 1 | 1 | 7 | 87.5 | **Low** |
|  | Siddique et al. (65) | 1 | 1 | 1 | 1 | 0 | 0 | 1 | 1 | 6 | 75 | **Low** |
|  | Sidra et al. (66) | 1 | 1 | 1 | 1 | 0 | 0 | 1 | 1 | 6 | 75 | **Low** |
|  | Zangeneh et al.(67) | 1 | 1 | 1 | 1 | 0 | 0 | 1 | 1 | 6 | 75 | **Low** |

***Q*** *– Question;* ***Q1*** *– Were the criteria for inclusion in the sample clearly defined?* ***Q2*** *– Were the study subjects and the setting described in detail?* ***Q3*** *– Was the exposure measured in a valid and reliable way?* ***Q4*** *– Were objectives standard criteria used for measurement of the condition?* ***Q5*** *– Were confounding factors identified?* ***Q6*** *– Were strategies to deal with confounding factors stated?* ***Q7*** *– Were the outcomes measured in a valid and reliable way?* ***Q8*** *– Was appropriate statistical analysis used?*
